# Variants in *BANK1* are associated with lupus nephritis of European ancestry

**DOI:** 10.1038/s41435-021-00142-8

**Published:** 2021-06-14

**Authors:** Karin Bolin, Juliana Imgenberg-Kreuz, Dag Leonard, Johanna K. Sandling, Andrei Alexsson, Pascal Pucholt, Malena Loberg Haarhaus, Jonas Carlsson Almlöf, Joanne Nititham, Andreas Jönsen, Christopher Sjöwall, Anders A. Bengtsson, Solbritt Rantapää-Dahlqvist, Elisabet Svenungsson, Iva Gunnarsson, Ann-Christine Syvänen, Karoline Lerang, Anne Troldborg, Anne Voss, Øyvind Molberg, Søren Jacobsen, Lindsey Criswell, Lars Rönnblom, Gunnel Nordmark

**Affiliations:** 1 Department of Medical Sciences and Science for Life Laboratory, Uppsala University, Uppsala, Sweden; 2 Department of Medicine Solna, Karolinska Institutet, Karolinska University Hospital Stockholm, Stockholm, Sweden; 3 Molecular Medicine, Department of Medical Sciences and Science for Life Laboratory, Uppsala University, Uppsala, Sweden; 4 Russell/Engleman Rheumatology Research Center, Department of Medicine, University of California San Francisco, San Francisco, CA USA; 5 Department of Rheumatology, Lund University, Lund, Sweden; 6 Department of Biomedical and Clinical Sciences, Linköping University, Linköping, Sweden; 7 Department of Public health and Clinical Medicine, Umeå University, Umeå, Sweden; 8 Department of Rheumatology, University of Oslo, Oslo, Norway; 9 Department of Rheumatology, Aarhus University Hospital and Department of Biomedicine, Aarhus University, Aarhus, Denmark; 10 Department of Rheumatology, Odense University Hospital, Odense, Denmark; 11 Department of Clinical Medicine, Copenhagen University Hospital, Copenhagen, Denmark

**Keywords:** Disease genetics, Genetic association study

## Abstract

The genetic background of lupus nephritis (LN) has not been completely elucidated. We performed a case-only study of 2886 SLE patients, including 947 (33%) with LN. Renal biopsies were available from 396 patients. The discovery cohort (Sweden, *n* = 1091) and replication cohort 1 (US, *n* = 962) were genotyped on the Immunochip and replication cohort 2 (Denmark/Norway, *n* = 833) on a custom array. Patients with LN, proliferative nephritis, or LN with end-stage renal disease were compared with SLE without nephritis. Six loci were associated with LN (*p* < 1 × 10^−4^, *NFKBIA, CACNA1S*, *ITGA1*, *BANK1, OR2Y,* and *ACER3*) in the discovery cohort. Variants in *BANK1* showed the strongest association with LN in replication cohort 1 (*p* = 9.5 × 10^−4^) and proliferative nephritis in a meta-analysis of discovery and replication cohort 1. There was a weak association between *BANK1* and LN in replication cohort 2 (*p* = 0.052), and in the meta-analysis of all three cohorts the association was strengthened (*p* = 2.2 × 10^−7^). DNA methylation data in 180 LN patients demonstrated methylation quantitative trait loci (meQTL) effects between a CpG site and *BANK1* variants. To conclude, we describe genetic variations in *BANK1* associated with LN and evidence for genetic regulation of DNA methylation within the *BANK1* locus. This indicates a role for *BANK1* in LN pathogenesis.

## Introduction

Systemic lupus erythematosus (SLE) is a complex autoimmune disease predominantly affecting women in their child-bearing age. Lupus nephritis (LN) constitutes one of the main clinical challenges in patients with SLE and is a cause of significant morbidity and mortality. LN occurs in 15–55% of patients with SLE with a higher incidence in Asian and African populations [1, 2]. Proliferative glomerulonephritis, defined as class III/IV according to the histopathological classification systems ISN/RPS 2003 or WHO, is considered the most severe form of nephritis and requires immunosuppressive treatment with glucocorticoids and mycophenolate mofetil or cyclophosphamide [3, 4]. Despite improved treatment regimens, approximately 10% of all LN patients develop end-stage renal disease (ESRD) [1].

The genetic background to SLE has been thoroughly investigated through candidate gene and genome-wide association studies. To date, more than 100 SLE risk loci have been identified that explain a significant proportion of SLE heritability [5]. Less is known about the genetic background of LN. Distinct genetic factors associated with LN in patients of different ethnicities have been reported [2]. Some of the known SLE susceptibility genes, which function in the immune system, seem to be also associated with LN. Still, more renal-specific genes predispose specifically to LN. Genetic variants in *HLA-DR, ITGAM, FCGR, IRF5, TNIP1, STAT4,* and *TNFSF4* have been associated with both SLE per se and with LN, whereas *APOL1, PDGFRA,* and *HAS2* have been identified in LN specifically [6]. Genetic variants in *STAT4* have been proposed to associate with SLE and LN in general, and with a more severe subtype of LN and renal failure [7, 8].

Epigenetic regulation, such as DNA methylation, has been proposed to be of importance in SLE pathogenesis [9]. Epigenetic mechanisms affect gene expression without altering the underlying DNA sequence. Hypomethylation of type I interferon-induced genes in patients with SLE has been well established [10, 11]. In LN, a role of epigenetic regulation has been suggested, e.g., the type I interferon regulator gene *IRF7* is differently methylated between SLE patients with and without renal involvement [12]. This case-only study aimed to further elucidate the genetic and epigenetic background to LN and its subtypes using data from three large SLE cohorts.

## Results

### Patient characteristics

Patient characteristics are described in Table 1. In all cohorts, patients with LN were more often men, younger at diagnosis, and presented with a higher number of American College of Rheumatology (ACR) criteria than SLE patients without LN. Renal biopsy data were available for the discovery and replication cohort 1, where proliferative nephritis was the most common class of nephritis (173/278, 62% in the discovery cohort and 65/118, 55% in replication cohort 1). Among LN patients with available renal function data at follow-up, 38/290 (13%) in the discovery cohort and 48/216 (22%) in replication cohort 1 proceeded to ESRD.

**Table 1:** Patient characteristics.

	Discovery cohort (Sweden)				Replication cohort 1 (USA)				Replication cohort 2 (Denmark/Norway)			
	SLE	Nephritis^a^	SLE without nephritis	*P* value^b^	SLE	Nephritis^a^	SLE without nephritis	*P* values^b^	SLE	Nephritis^a^	SLE without nephritis	*P* value^b^
Number of patients (%)	1091	377 (34.6)	714 (65.4)		962	216 (22.5)	746 (77.5)		833	354 (42.5)	479 (57.5)	
Number of females (%)	941 (86.3)	290 (76.9)	651 (91.2)	<0.001	883 (91.8)	184 (85.2)	699 (93.7)	<0.001	746 (89.6)	305 (86.2)	441 (92.1)	<0.01
Age at SLE diagnosis, years (mean ± SD)	35.8 ± 15.9	30.7 ± 15.0	38.5 ± 15.6	<0.001	35.3 ± 13.4	28.2 ± 13.3	37.3 ± 12.7	<0.001	32.6 ± 13.5	29 ± 12.9	35.2 ± 13.4	<0.001
Disease duration, years (mean ± SD)	22.3 ± 12.0	23.6 ± 11.7	21.5 ± 12.0	<0.01	9.19 ± 8.63	12.3 ± 9.6	8.3 ± 8.1	<0.001	16.6 ± 10.7	17.1 ± 11.2	16.3 ± 10.4	0.29
ACR criteria (mean ± SD)	5.6 ± 1.4	6.2 ± 1.5	5.3 ± 1.2	<0.001	4.9 ± 1.5	5.9 ± 1.7	4.6 ± 1.3	<0.001	5.9 ± 1.4	6.5 ± 1.6	5.6 ± 1.2	<0.001
Class I/II nephritis^c^, *n* (%)		38/278 (13.7)										
Class III/IV proliferative nephritis^d^, *n* (%)		173/278 (62.2)				65/118 (55.1)						
Class V nephritis^e^, *n* (%)		48/278 (17.3)										
ESRD^f^, *n* (%)		38/290 (13.1)				48/216 (22.2)						

^a^Defined according to the ACR nephritis criteria or as a biopsy confirmed LN in the presence of ANA or anti-dsDNA antibodies, according to the SLICC classification.

^b^*P* value for the difference between nephritis and SLE without nephritis. Categorical variables were compared with the χ2 test and continuous variables by student’s unpaired *t*-test.

^c^WHO or ISN/RPS 2003 class I or II only, among patients who had undergone renal biopsy and for whom biopsy results were available.

^d^WHO or ISN/RPS 2003 class III or IV ever, among patients who had undergone renal biopsy and for whom biopsy results were available.

^e^WHO or ISN/RPS 2003 pure class V, among patients who had undergone renal biopsy and for whom biopsy results were available.

^f^Dialysis or transplantation, among patients with available follow-up data on renal function.

A total of 19/278 patients with available biopsy results had histopathological findings other than a typical LN pattern and classification according to the WHO or ISN/RPS classification systems were not possible.

### Genetic association analysis

#### Discovery cohort

**Fig. 1:**
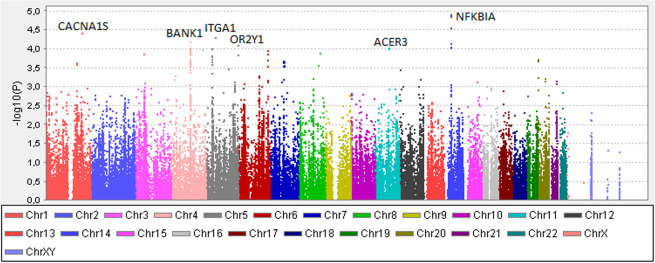
Association between 112,815 SNPs and lupus nephritis in the discovery cohort. Manhattan plot displaying results from the association analysis of 112,815 SNPs in 377 patients with lupus nephritis (LN) and 714 SLE without nephritis in the discovery cohort. The negative logarithm of the *p*-value is plotted against the chromosomal location of the tested variants. Genes with SNPs associated with LN with *p* < 1 × 10^−4^ are denoted.

First, we compared allele frequencies between SLE patients with (*n* = 377) and without LN (*n* = 714) in the discovery cohort. The strongest signals of association with LN were found for four highly-linked single nucleotide polymorphisms (SNPs) close to the Nuclear Factor of Kappa Light Polypeptide Gene Enhancer in B Cells Inhibitor, Alpha (*NFKBIA)* gene (top SNP rs12433012, *p* = 1.3 × 10^−5^, OR: 0.54, 95% CI: 0.41–0.71). A total of 139 SNPs were associated with *p* < 0.001. Associations with *p* < 1 × 10^−4^ were observed for SNPs in Calcium Voltage-Gated Channel Subunit Alpha1 S (*CACNA1S)*, Integrin Subunit Alpha 1 (*ITGA1)*, B Cell Scaffold Protein with Ankyrin Repeats 1 (*BANK1)*, Olfactory Receptor Family 2 Subfamily Y Member 1 (*OR2Y1)* and Alkaline Ceramidase 3 *(ACER3)* (Fig. 1, Supplementary Table 1).

We then analyzed the association between genetic variants and the subgroup proliferative nephritis, comparing LN patients with proliferative nephritis (*n* = 173) versus SLE without nephritis (*n* = 714). Associations with *p* < 1 × 10^−4^ were found between proliferative nephritis and SNPs in *ITGA1* and *BANK1* (Supplementary Table 2). Finally, analysis was performed comparing LN patients who had reached ESRD (*n* = 38) versus SLE without nephritis (*n* = 714). An association between LN patients with ESRD and rs2763321 in the Membrane Palmitoylated Protein 7 (*MPP7)* gene was found (*p* = 7.4 × 10^−6^) (Supplementary Table 3).

#### Replication cohort 1

All SNPs reaching a *p*-value of <0.001 in the analyses of LN (*n*_SNPs_ = 139), proliferative nephritis (*n*_SNPs_ = 198), or ESRD (*n*_SNPs_ = 182) versus SLE without nephritis in the discovery cohort were analyzed in replication cohort 1. In the analysis of LN (*n* = 216) versus SLE without nephritis (*n* = 746), the strongest signal of association was identified for a SNP in *BANK1* (*p* = 9.5 × 10^−4^) (Table 2). When analyzing proliferative nephritis (*n* = 65) and ESRD (*n* = 48) versus SLE without nephritis, no associations with *p* < 0.001 were found (data not shown).

**Table 2:** Association analysis of patients with LN versus SLE without nephritis in the discovery cohort and replication cohort 1 and meta-analysis.

					Discovery cohort, Sweden				Replication cohort 1, USA				Meta- analysis
CHR	SNPs	Minor allele	Major allele	Gene	MAF^LN+^	MAF^LN−^	OR (95% CI)	*P*	MAF^LN+^	MAF^LN−^	OR (95% CI)	*P*	*P*
4	rs4699261	A	G	*BANK1*	0.22	0.30	0.66 (0.54–0.81)	9.9 × 10^−5^	0.24	0.33	0.65 (0.50–1.19)	9.5 × 10^−4^	***3.3*** **×** ***10***^*−7*^
4	rs4699259	A	C	*BANK1*	0.22	0.30	0.66 (0.54–0.81)	9.8 × 10^−5^	0.24	0.32	0.65 (0.50–1.19)	1.2 × 10^−3^	***4.2*** **×** ***10***^*−7*^
4	rs34851381	G	A	*BANK1*	0.22	0.30	0.66 (0.54–0.81)	9.9 × 10^−5^	0.24	0.33	0.66 (0.51–0.85)	1.4 × 10^−3^	***4.8*** **×** ***10***^*−7*^
4	rs17266357	G	A	*BANK1*	0.22	0.30	0.66 (0.54–0.81)	9.9 × 10^−5^	0.24	0.33	0.66 (0.51–0.85)	1.5 × 10^−3^	***5.0*** **×** ***10***^*−7*^
4	rs35194352	G	A	*BANK1*	0.22	0.30	0.66 (0.54–0.81)	9.9 × 10^−5^	0.24	0.33	0.66 (0.51–0.85)	1.4 × 10^−3^	***5.0*** **×** ***10***^*−7*^
4	rs13146194	G	A	*BANK1*	0.22	0.30	0.66 (0.54–0.81)	9.9 × 10^−5^	0.24	0.33	0.66 (0.51–0.85)	1.5 × 10^−3^	***5.0*** **×** ***10***^*−7*^
4	rs11929782	G	A	*BANK1*	0.22	0.30	0.66 (0.54–0.81)	9.9 × 10^−5^	0.24	0.33	0.66 (0.51–0.85)	1.5 × 10^−3^	***5.1*** **×** ***10***^*−7*^
4	rs34499378	A	G	*BANK1*	0.22	0.30	0.67 (0.54–0.82)	1.3 × 10^−4^	0.24	0.33	0.66 (0.51–0.85)	1.3 × 10^−3^	***5.9*** **×** ***10***^*−7*^
4	rs11940244	A	C	*BANK1*	0.22	0.30	0.66 (0.54–0.82)	1.1 × 10^−4^	0.24	0.33	0.66 (0.51–0.85)	1.6 × 10^−3^	***5.9*** **×** ***10***^*−7*^
4	rs10446708	G	A	*BANK1*	0.22	0.30	0.66 (0.54–0.81)	9.9 × 10^−5^	0.24	0.32	0.67 (0.52–0.86)	1.8 × 10^−3^	***6.1*** **×** ***10***^*−7*^
4	rs35838403	A	G	*BANK1*	0.22	0.30	0.66 (0.54–0.82)	1.1 × 10^−4^	0.24	0.33	0.67 (0.52–0.85)	1.8 × 10^−3^	***6.8*** **×** ***10***^*−7*^
4	rs66638185	G	A	*BANK1*	0.22	0.30	0.65 (0.53–0.82)	6.7 × 10^−5^	0.23	0.32	0.64 (0.48–1.37)	3.1 × 10^−3^	***6.9*** **×** ***10***^*−7*^
4	rs35416717	A	C	*BANK1*	0.22	0.30	0.66 (0.54–0.82)	1.1 × 10^−4^	0.24	0.33	0.67 (0.52–0.85)	1.9 × 10^−3^	***7.0*** **×** ***10***^*−7*^
4	rs11931087	G	A	*BANK1*	0.22	0.30	0.66 (0.54–0.82)	1.1 × 10^−4^	0.24	0.33	0.67 (0.52–0.85)	1.9 × 10^−3^	***7.0*** **×** ***10***^*−7*^
4	rs10446682	A	G	*BANK1*	0.22	0.30	0.67 (0.54–0.82)	1.3 × 10^−4^	0.24	0.33	0.67 (0.52–0.86)	1.7 × 10^−3^	***7.3*** **×** ***10***^*−7*^
4	rs4699262	A	G	*BANK1*	0.22	0.30	0.66 (0.54–0.82)	1.1 × 10^−4^	0.24	0.33	0.67 (0.52–1.19)	2.0 × 10^−3^	***7.4*** **×** ***10***^*−7*^
4	rs17266552	A	G	*BANK1*	0.22	0.30	0.66 (0.54–0.82)	1.1 × 10^−4^	0.24	0.33	0.67 (0.52–0.85)	2.0 × 10^−3^	***7.4*** **×** ***10***^*−7*^
4	rs17200433	G	A	*BANK1*	0.22	0.30	0.66 (0.54–0.81)	1.1 × 10^−4^	0.23	0.30	0.65 (0.49–0.85)	2.6 × 10^−3^	***8.7*** **×** ***10***^*−7*^
4	rs13117264	C	A	*BANK1*	0.23	0.30	0.67 (0.54–0.82)	1.2 × 10^−4^	0.25	0.33	0.67 (0.52–0.85)	2.2 × 10^−3^	***9.0*** **×** ***10***^*−7*^
4	rs34261083	A	G	*BANK1*	0.22	0.29	0.67 (0.54–0.83)	1.9 × 10^−4^	0.24	0.32	0.66 (0.51–0.85)	1.4 × 10^−3^	***9.1*** **×** ***10***^*−7*^

Discovery cohort: LN, *n* = 377; SLE without nephritis, *n* = 714. Replication cohort 1: LN, *n* = 216; SLE without nephritis, *n* = 746. *MAF* minor allele frequency, *LN* lupus nephritis, *OR* odds ratio, *CI* confidence interval. Numbers in bold italic are significant after Bonferroni correction for 48,000 independent SNPs on the Immunochip, *p* < 1.0 × 10^−6^. Total analyzed SNPs, *n* = 112 815.

**Fig. 2:**
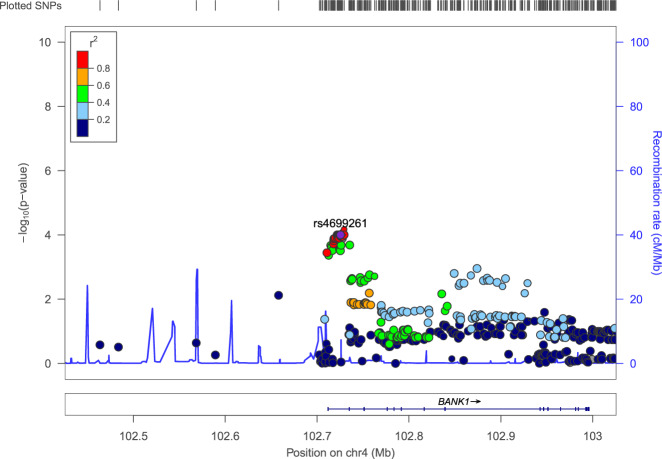
Regional association plot of the *BANK1* region. Regional association plot of the *BANK1* region displaying results from the analysis of LN (*n* = 377) versus SLE without nephritis (*n* = 714) in the discovery cohort. Top SNP rs4699261 is in strong linkage disequilibrium (*r*² ≥ 0.8) with a cluster of SNPs located in the first intronic region.

Meta-analyses were performed using the results from the discovery and replication cohort 1 for LN, proliferative nephritis, and ESRD versus SLE without nephritis, respectively. In the LN meta-analysis, the strongest signal of association was found for several highly-linked SNPs in *BANK1* (*p*_meta_ = 3.3 × 10^−7^ for the top SNP rs4699261), passing the Bonferroni-corrected *p*-value of <1.0 × 10^−6^ (Table 2 and Supplementary Fig. 1). A regional association plot of the *BANK1* region based on the analysis of LN versus SLE without nephritis in the discovery cohort, revealed a cluster of highly linked SNPs in the first intron region (Fig. 2).

In the meta-analysis of proliferative nephritis versus SLE without nephritis, associations were observed for several SNPs in *BANK1* (top SNP rs6856202, *p*_meta_ = 1.3 × 10^−5^, *r*^2^ = 0.53 to rs4699261, Supplementary Table 4. Finally, a meta-analysis of ESRD versus SLE without nephritis found an association for rs12573804 in *long intergenic non-protein coding RNA 1515* (*LINC01515)* on chromosome 10 (*p*_meta_ = 9.6 × 10^−6^, Supplementary Table 5).

#### Replication cohort 2

Ten SNPs from genes with the highest signals of association to LN in the discovery cohort were selected and successfully genotyped in replication cohort 2, consisting of 833 patients with SLE from Denmark and Norway. In the analysis of patients with LN (*n* = 354) versus SLE without nephritis (*n* = 479), no significant associations between these SNPs and LN were found (Supplementary Table 6). However, *BANK1* SNP rs4699259 (*r*^2^ = 0.98 to rs4699261) was associated with LN with *p* = 0.052 and the same direction of effect (OR: 0.80, 95% CI: 0.64–1.0) as in the discovery cohort and replication cohort 1. A random-effect meta-analysis was performed using the results for rs4699259 from all three cohorts. In this meta-analysis, the association between LN and *BANK1* was strengthened (*p*_meta_ = 2.2 × 10^−7^).

#### Case-control analysis

To further corroborate the association between LN and *BANK1*, the top SNP rs4699261 was investigated in a case-control analysis of patients from the discovery cohort with SLE (*n* = 1091) and stratified for SLE without nephritis (*n* = 714) and LN (*n* = 377) versus healthy controls (*n* = 2707), respectively. An association with SLE was found with *p* = 2.0 × 10^−4^ and OR 0.80 (95% CI: 0.71–0.90). In the analysis of SLE without nephritis versus controls, no association with BANK1 was found (*p* = 0.28, OR 0.93, 95% CI: 0.81–1.07). When comparing LN patients with controls, a stronger association with *BANK1* SNP rs 4699261 could be determined with *p* = 6.0 × 10^−7^ and OR 0.62 (95% CI: 0.52–0.75).

### Analysis of genetic regulation of DNA methylation by LN associated variants

**Fig. 3:**
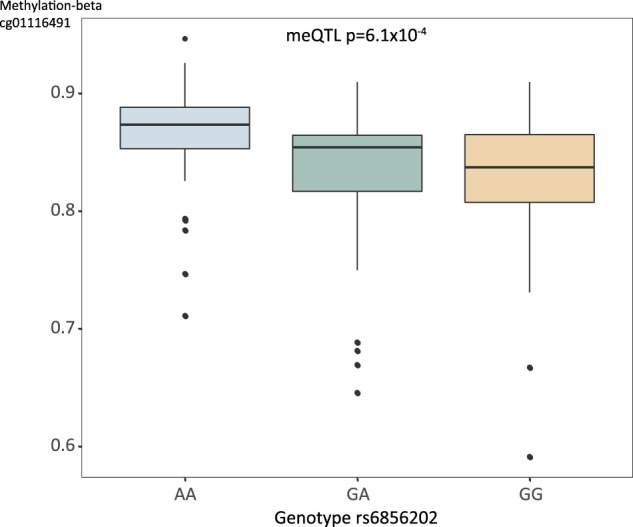
Genetic regulation of methylation in lupus nephritis at BANK1. Box plot of the *BANK1* meQTL rs6856202−cg01116491. SNP genotypes at rs6856202 are shown on the *x*-axis, methylation-beta values of CpG site cg01116491 are shown on the *y*-axis. The major allele (A) of rs6856202 is associated with increased methylation at cg01116491 (*p*_meQTL_ = 6.0 × 10^−4^) in whole blood from patients with LN. The major allele (A) is the risk allele for LN in the discovery cohort and displayed the highest signal of genetic association to proliferative nephritis in the meta-analysis of discovery and replication cohort 1 (*p*_meta_ = 1.3 × 10^−5^). Box plot center lines indicate medians, box boundaries indicate first and third quartile, and whiskers extend to data points located within 1.5 times the length of interquartile range from the median.

Next, we aimed to investigate whether the effects of LN-associated genetic variants from this and previous studies, could be mediated through changes in DNA methylation. A methylation quantitative trait loci (meQTL) analysis was performed in whole blood from 180 LN patients from the discovery cohort against the genotypes. The strongest meQTL effects were identified in Integrin Subunit Alpha M *(ITGAM)* and B lymphocyte kinase (*BLK)*. However, genetic variants in these genes were not significantly associated with LN in this study (data not shown). In the *BANK1* locus, we identified meQTL effects between CpG site cg01116491 and several SNPs (top SNP rs6856202, *p*_meQTL_ = 6.1 × 10^−4^, *r*^2^ = 0.52 to rs4699259, Supplementary Table 7). The SNP rs6856202 major allele (A) is the risk allele, associated with LN in the discovery cohort (*p* = 3.1 × 10^−4^) and with proliferative nephritis in the meta-analysis of discovery and replication cohort 1 (*p* = 1.3 × 10^−5^) (Supplementary Tables 1, 4). LN patients homozygous for the risk allele (A) showed hypermethylation at CpG site cg01116491 compared with LN patients heterozygous (GA) or homozygous for the non-risk allele (G) (Fig. 3). Association with DNA methylation levels was also found for another block of SNPs in the *BANK1* locus (top SNP rs7683892, *r*^2^ = 0.57 to rs4699259). However, we did not observe a direct effect of the top SNP rs4699259 with the level of DNA methylation at any *BANK1* CpG site (data not shown).

### Functional annotation of associated variants

To further investigate the potential functional role of the identified variants, public databases were queried. In Open Targets Genetics [13], SNP rs4699259 is shown to be an expression quantitative trait locus (eQTL) for *BANK1* in lymphoblastoid cell lines, where the risk allele (C) is associated with upregulated *BANK1* mRNA expression (*β* = 0.20, *p* = 5.8 × 10^−10^). Furthermore, in the GTEx portal, rs4699259 is proposed to be an eQTL for *BANK1* expression in subcutaneous adipose tissue, and in HaploReg v4.1, rs4699262 (in perfect LD with rs4699259, *r*^2^ = 1.0) is shown to be an eQTL for *BANK1* expression in the blood (*p* = 5.7 × 10^−5^) [14].

## Discussion

Here we present associations between SNPs in *BANK1* and LN in the hitherto most extensive case-only study, comprising nearly 3000 patients with SLE of European ancestry. Furthermore, to the best of our knowledge, this is the first time *BANK1* SNPs have been associated with the severe form of proliferative nephritis. We also present novel evidence for genetic regulation of DNA methylation within the *BANK1* locus in patients with LN. The top SNP is an eQTL for *BANK1,* where the risk allele is associated with upregulated *BANK1* mRNA expression in lymphoblastoid cell lines.

To investigate potential functional effects of *BANK1* variants, we performed a meQTL analysis using whole blood from patients with LN. The *BANK1* top SNP associated with proliferative nephritis displayed a meQTL effect, where LN patients homozygous for the risk genotype showed hypermethylation at CpG site cg0111649. The CpG site cg01116491 is located in a gene body region approximately 3300 bp downstream of the transcription start site of *BANK1*. Hypermethylation in gene body regions can be an indication of increased gene expression [15, 16]. This meQTL site is in a region that overlaps with histone modification mark H3K36me3 in B cells, and H3K36me3 is associated with actively transcribed gene bodies [17]. These findings could indicate an effect of the risk genotype for *BANK1* in upregulating *BANK1* expression, mediated by epigenetic regulation.

Searching databases, SLE-associated variants in *BANK1* are eQTLs in multiple tissues, where the risk alleles associate with increased *BANK1* expression [18]. Interestingly, different eQTL effects for *BANK1* SNP rs4637409 (*r*^2^ = 0.75 to rs4699261) in B cells from healthy males and females have been described [19]. In males, the SLE risk allele was associated with increased expression of the *SLC39A8* gene, located downstream of *BANK1*, whereas the eQTL effect was opposite in females. Sex-biased eQTL effects are intriguing since LN is more common in male patients with SLE, and this topic warrants further investigations.

BANK1 is a B cell adaptor protein primarily expressed in mature B cells and, to a lesser extent, in myeloid and plasmacytoid dendritic cells [18, 20]. Upon B cell receptor activation, BANK1 enhances calcium mobilization, becomes tyrosine phosphorylated, and can promote Lyn-mediated phosphorylation of inositol 1,4,5-trisphosphate receptors (IP3R) [20]. BANK1 is 785aa in its full-length (FL) form and has a smaller isoform lacking the second exon (D2 isoform), which encodes a Toll/IL-1 receptor domain (TIR) [21]. The SLE risk variant rs10516487 in exon 2, first described by Kozyrev et al., is causing a nonsynonymous substitution (R61H) and correlates with decreased splicing of exon 2 and a higher expression of the FL isoform containing the TIR domain [21, 22]. BANK1 is functionally linked to Toll-like receptor (TLR) pathways with downstream activation of transcription factor NFκB and IFN regulatory factors, promoting B cell activation and inflammation, and prominent features of SLE [23–26].

Genetic variations in *BANK1* have convincingly been associated with SLE and replicated in multiple ethnicities [18, 21, 27–30]. The often investigated exon 2 non-synonymous variant rs10516487 (R61H), displays associations with SLE of approximately the same magnitude in all studied ethnicities (≈OR 0.70 referring to the minor allele), despite differences in minor allele frequency among control populations. Martinez-Bueno et al. reported a trans-ethnic mapping of *BANK1* associations with SLE in Europeans and African−Americans [18]. The associated markers covered the same regions in both populations but haplotype blocks differed, with greater diversity among African−Americans. We conclude that although there are trans-ethnic differences in haplotype structure, the association between SLE and *BANK1* variants is robust across ethnicities.

Stratifying SLE patients for the immunologic, hematological, or renal ACR criteria or presence of anti-dsDNA antibodies, more robust associations with *BANK1* have been reported compared with analyses of all SLE patients versus controls [28, 31]. Several autoimmune diseases report associations with *BANK1*, including systemic sclerosis, rheumatoid arthritis, autoimmune thyroid disease, and germinal center formation in Sjögren’s syndrome minor salivary glands, suggesting *BANK1* as a general autoimmunity susceptibility gene [32–35]. The exon 2 variant rs10516487 (*r*^2^ = 0.75 to rs4699259) was only weakly associated with LN in our case-only analysis of the discovery cohort (OR 0.76, 95% CI 0.61–0.95, *p* = 0.015, data not shown) and did not pass our filtering criteria for analysis in replication cohorts 1 and 2. The exon 2 variant may confer risk for SLE per se, whereas the intron 1 variants here described having an impact on disease severity such as LN, particularly the more severe subtype proliferative nephritis.

While LN case-control analyses inherently also are SLE case-control studies, we chose to perform and replicate a case-only study comparing SLE patients with or without LN in an attempt to refine the genetic contribution to LN. To further determine if the *BANK1* association was primarily with LN and not SLE per se, we performed a post hoc case-control analysis of the discovery cohort comparing SLE, SLE without nephritis, and LN versus controls, respectively. We found the association with *BANK1* to be stronger in the analyses, LN versus controls and LN versus SLE without nephritis (Table 2), than the SLE case-control and SLE without nephritis-control analyses, and conclude the association with *BANK1* is mainly with LN. Chung et al. performed a case-only meta-analysis of three genome-wide association studies (GWAS) and found an association with LN within the PDGF receptor-α (*PDGFRA*) gene locus [36]. Unfortunately, this gene locus was not covered on the Immunochip used in this study. In our previous study, the *STAT4* SNP rs7582694 was associated with LN with severe renal insufficiency (glomerular filtration rate <30 mL/min/1.73m^2^) [7]. The outcome of severe renal insufficiency was not analyzed in the current study. However, *STAT4* SNP rs10181656 (*r*^2^ = 1.0 to rs7582694) displayed a trend for association with ESRD (*p* = 0.07, data not shown).

Interestingly, in concordance with previous observations, the highest signals of association with LN were assigned to genes outside the MHC locus [7, 37]. The conclusion is that although variants in the MHC locus confer the strongest genetic risk for SLE, other gene regulatory regions are of importance in the development of LN. The genetic risk for LN has also been assessed by calculating genetic risk scores (GRS). Reid et al. concluded that SLE patients with a high GRS were at increased risk of developing renal disorder, including proliferative nephritis and ESRD [38]. Webber et al. found a high GRS to be associated with proliferative nephritis and a greater risk for LN in childhood than in adult SLE [37]. Both of these studies included *BANK1* variants in their GRS.

The importance of B cells in LN pathogenesis and inflammation is not well defined. Infiltrating B cells can be found in more than half of LN biopsies, mostly in the tubulointerstitial compartment [39]. Upregulated mRNA expression of *BANK1* has been detected in renal biopsies from LN patients, mainly in the tubulointerstitial compartment [36]. Together with the genetic association between *BANK1* and LN presented here, a role for B cells in LN pathogenesis is suggested. Single-cell RNA sequencing of samples from LN biopsies has determined different B cell clusters with upregulated genes defining an activated B cell state [40]. Despite this, trials with anti-B cell therapy in LN have shown varying success [41, 42]. The use of genetic and other biomarkers may improve patient stratification for treatment decisions in the future [43].

The strengths of this study are the replication of our findings in large cohorts of well classified SLE patients with homogenous genetic backgrounds. Renal biopsy data could be retrieved from two of the cohorts, making it possible to assess the subgroup with proliferative nephritis. The availability of DNA methylation data from our discovery cohort adds an epigenetic layer to our genetic findings. A limitation is a cross-sectional design, where follow-up time varied between patients. Some SLE patients without nephritis may develop LN in the future and the subtype may also change during follow-up. This study only involved SLE patients of European ancestry, and *BANK1* associations to LN in other ethnicities remain to be studied.

To conclude, we here demonstrate an association between *BANK1* and LN in three large cohorts of SLE patients of European ancestry. Furthermore, evidence of genetic control of methylation levels within the *BANK1* locus was observed. The upregulation of *BANK1* gene expression in renal biopsies from LN patients indicates a functional role of *BANK1*, although the exact mechanisms in LN pathogenesis need to be further elucidated.

## Subjects and Methods

### Patients

#### Discovery cohort

The discovery cohort included 1155 Swedish SLE patients, out of which 1091 passed genotype quality control (QC). The patients originated from Stockholm (*n* = 346), Linköping (*n* = 172), Uppsala (*n* = 188), Lund (*n* = 153), and Umeå (*n* = 232), and fulfilled either the 1982 ACR criteria for SLE or had a biopsy confirmed LN in the presence of anti-nuclear antibodies (ANA) or anti-double-stranded DNA (dsDNA) antibodies, according to the SLICC classification [44, 45]. Renal biopsy histopathology was available from 278/377 (73.7%) patients. The study protocol was approved by the local ethics committees, and the patients gave written informed consent.

#### Replication cohort 1

Replication cohort 1 included 962 patients after QC, with SLE from the University of California, San Francisco (UCSF) Lupus Genetics project [46]. European ancestry was determined using STRUCTURE, including individuals being ≥85% European [47]. All patients completed an extensive questionnaire and the SLE diagnosis was confirmed by medical record review according to the ACR criteria [45]. Renal biopsy histopathology was available from 118/216 (54.6 %) patients. The study protocol was approved by the local ethics committees, and the patients gave written informed consent.

#### Replication cohort 2

Replication cohort 2 consisted of 854 patients with SLE from Denmark and Norway, all of self-reported European origin and fulfilling ≥4 ACR criteria for SLE [45]. After QC, 833 patients remained for analysis. The study protocol was approved by the local ethics committees, and the patients gave written informed consent. Patient characteristics are shown in Table 1.

#### Controls

Healthy blood donors were recruited as previously described [48]. After QC, 2707 healthy controls remained.

### Genotyping and quality control

The discovery cohort and controls were genotyped on the Illumina Infinium Immunochip (San Diego, California, USA), containing 196,524 SNPs covering the major autoimmune diseases [49]. Genotyping was performed at the SNP&SEQ Technology Platform, part of the National Genomics Infrastructure (NGI) at Uppsala University, Sweden. SNP-based QC filters were call rate >95%, Hardy−Weinberg equilibrium (HWE) *p* > 1 × 10^−6^, and MAF > 0.05. Sample QC included principal component analysis (PCA) performed on 1000 Genomes Project data, where individuals of non-European ancestry were removed, cryptic relatedness analysis with the removal of second-degree relatives or closer, and autosomal heterozygosity with the removal of individuals with anomalously high (*F* ≤ −0.1) or low (*F* ≤ 0.1) heterozygosity. The sample call rate was set to 96%. After QC, 112,815 SNPs remained for analysis.

Replication cohort 1 was genotyped on the Immunochip in four different laboratories. The following QC was performed on the genotyping data from each lab in the following order prior to merging data from all four labs: (1) Removal of individuals with <80% complete genotyping, (2) removal of SNPs with <95% call rate, and (3) removal of individuals with <95% complete genotyping. Duplicates and first-degree relatives were removed by identity-by-descent analysis [50]. Ancestry outliers with substantial non-European ancestry were identified via EIGENSTRAT (>4SD in top three principal components) and excluded [51]. SNPs with HWE *p* < 1 × 10^−5^ (based on European controls) were removed. After QC, 128,263 SNPs remained for analysis.

A total of 15 SNPs with association to LN in the discovery cohort (all *p* < 0.0002) (Supplementary Table 1) and annotated in or nearby a gene were selected for genotyping in replication cohort 2. One SNP per gene in *NFKBIA, CACNA1S*, *PALLD*, *LOC10537883, ENPP2,* and *PKHD1L1* and two SNPs in *BANK1* and *ITGA1* were successfully genotyped. Replication cohort 2 was genotyped using the iPLEX chemistry on a MassARRAY system (Agena Bioscience). QC included a minimum SNP and individual call rate of 90%. Variants with differential missingness (*p* < 0.01) or HWE (*p* < 0.01, in controls) were excluded. Ten SNPs and 833 patients passed QC and had available information on LN occurrence according to ACR criteria (Denmark *n* = 547, Norway *n* = 286) [45].

The regional association plot was generated in LocusZoom software (Fig. 2). Linkage disequilibrium (LD) *r*^2^ values were derived from the 1000 Genomes Project CEU population (Northern Europeans from Utah) and extracted from the LDlink service [52] using the LDlinkR package [53] (Supplementary Fig. 1).

### Methylation quantitative trait loci (meQTL) analysis

We performed a *cis*-meQTL analysis investigating DNA methylation levels in whole blood from LN patients from the discovery cohort (*n* = 180 with DNA methylation data available) against the genotypes of SNPs with a nominal *p*-value <0.001 in LN versus SLE without nephritis analysis in the discovery cohort (*n* = 110 SNPs) and SNPs in genes that had been previously associated to LN (*n* = 482, *n* = 592 SNPs in total) [6]. DNA methylation data were generated on the HM450k methylation array, normalized and quality controlled as previously described [11]. All CpG sites located within a 100 kb flanking region of these SNPs were included, and methylation levels were tested in PLINK for genotype association in LN patients assuming an additive model.

### Definitions

LN was defined according to the ACR nephritis criteria or a biopsy confirmed LN in the presence of ANA or anti-dsDNA antibodies, according to the SLICC classification [44, 45]. Biopsies were classified according to the WHO or ISN/RPS 2003 classification systems [3]. Proliferative nephritis was defined as class III or IV nephritis in either classification system. Data on renal function were collected from patient charts at the latest available time of sampling, and glomerular filtration rate (GFR) was calculated using the modification of diet in renal disease study (MDRD) formula [54]. Patients with LN and ESRD, defined as either dialysis or transplantation, were identified.

### Statistical analysis

Allele frequencies were compared between patients with LN, proliferative nephritis, and ESRD respectively, using SLE patients without LN as a control group. Logistic regression was performed in PLINK software v 1.07 [50]. Sex and disease duration were included as covariates.

All SNPs with a nominal *p*-value of <0.001 in LN versus SLE without nephritis (*n* = 139 SNPs), proliferative nephritis versus SLE without nephritis (*n* = 198 SNPs), and ESRD versus SLE without nephritis (*n* = 182 SNPs) analyses in the discovery cohort were selected for analysis in replication cohort 1. Logistic regression was performed in PLINK, adjusting for sex, disease duration, and the first principal component for population stratification. Meta-analyses of the discovery cohort and replication cohort 1 were performed for LN, proliferative nephritis, and ESRD versus SLE without nephritis patients, using all SNPs that passed QC in both cohorts. Random-effects meta-analysis *p*-values are reported. A Bonferroni corrected *p*-value of <1.0 × 10^−6^ adjusting for 48,000 independent SNPs on the Immunochip was considered significant.

In replication cohort 2, logistic regression was performed in PLINK for patients with LN (*n* = 354) versus SLE without nephritis patients (*n* = 479). Sex and disease duration were used as covariates. A meta-analysis was performed using the logistic regression analyses of LN versus SLE without nephritis patients from all three cohorts. Random-effects meta-analysis *p*-values are reported.

The top SNP from association analysis of LN vs SLE patients without LN in the discovery cohort was further investigated in case-control analyses of patients with SLE and LN vs healthy controls (*n* = 2707), respectively. Allele frequencies were compared between patients and controls in a logistic regression analysis with age and sex as covariates.

## Supplementary information

Supplementary Figure 1.

Legend Supplementary Figure 1.

Supplementary tables 1-7
