# Supplementary figures and images for: Variants in BANK1 are associated with lupus nephritis of European ancestry
Source: Genes Immun. 2021 Jun 14;22(3):194–202. doi: 10.1038/s41435-021-00142-8 (PMC8277572; doi:10.1038/s41435-021-00142-8)

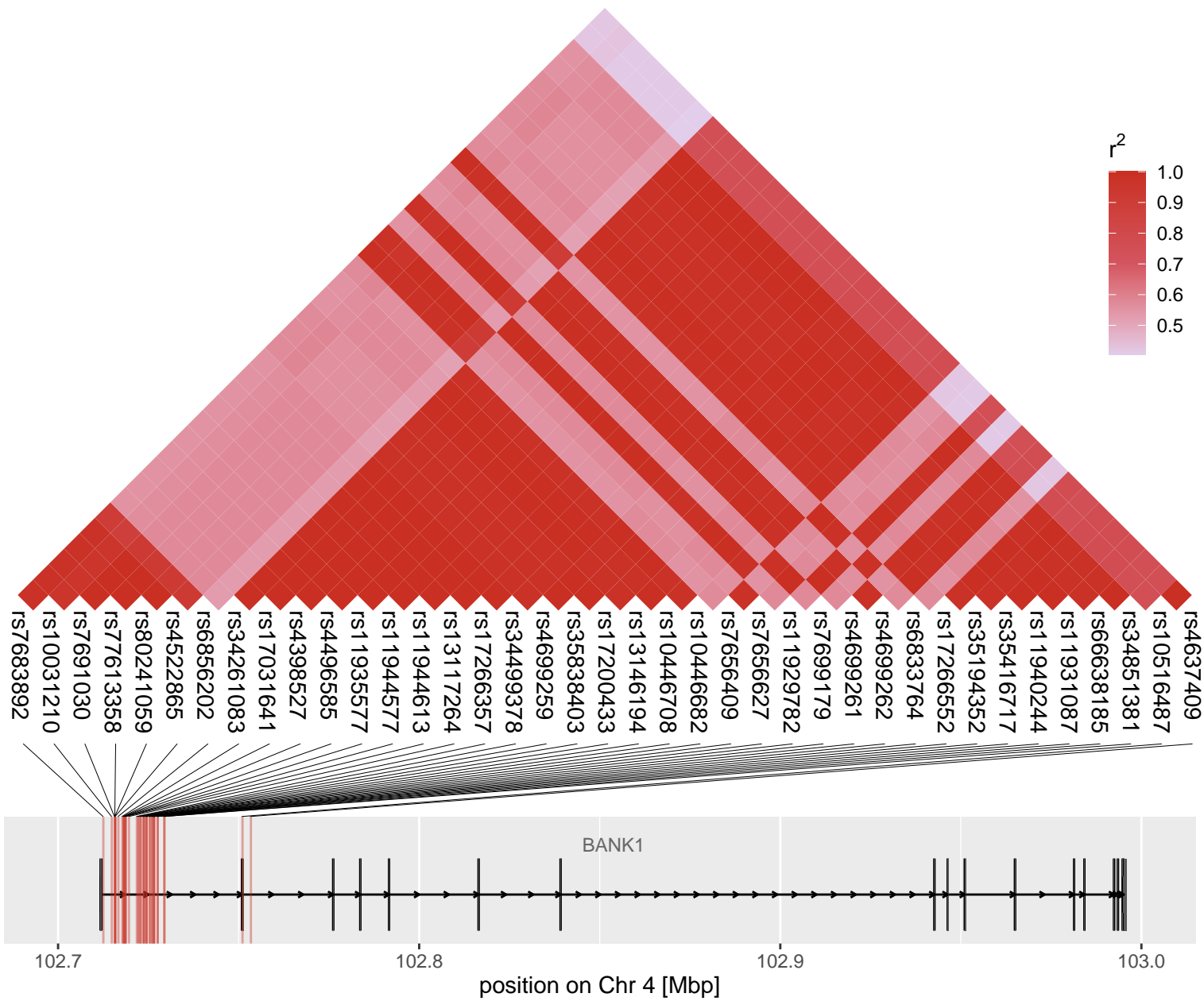

Supplement: Supplementary file 1 — Supplementary Figure 1. [file 41435_2021_142_MOESM1_ESM.pdf]
