# Supplementary material for: Variants in BANK1 are associated with lupus nephritis of European ancestry
Source: Genes Immun. 2021 Jun 14;22(3):194–202. doi: 10.1038/s41435-021-00142-8 (PMC8277572; doi:10.1038/s41435-021-00142-8)
Supplement: Supplementary file 2 — Legend Supplementary Figure 1. [file 41435_2021_142_MOESM2_ESM.docx]

**Supplementary Figure 1. Linkage disequilibrium plot of BANK1 SNPs**

Pairwise linkage disequilibrium (LD) among variants of interest and their location within the *BANK1* gene. LD is given as r² in the CEU part of the 1000 Genomes Project and was extracted from the LDlink service using the LDlinkR package.
