# Supplementary material for: Variants in BANK1 are associated with lupus nephritis of European ancestry
Source: Genes Immun. 2021 Jun 14;22(3):194–202. doi: 10.1038/s41435-021-00142-8 (PMC8277572; doi:10.1038/s41435-021-00142-8)
Supplement: Supplementary file 3 — Supplementary tables 1-7 [file 41435_2021_142_MOESM3_ESM.pdf]

Supplementary table 1. Association analysis of patients with LN vs SLE without nephritis in the discovery cohort

CHR	SNP	GENE	Minor allele	Major allele	MAF^{LN+}	MAF^{LN-}	OR (95% CI)	P
14	rs12433012	*NFKBIA**	A	G	0.11	0.18	0.54 (0.41-0.71)	$1.3x10^{-5}$
14	rs56173145	*NFKBIA**	A	G	0.11	0.18	0.54 (0.41-0.72)	$1.4x10^{-5}$
14	rs7155561	*NFKBIA**	C	G	0.11	0.18	0.56 (0.43-0.73)	$2.8x10^{-5}$
14	rs7155714	*NFKBIA**	A	G	0.11	0.18	0.56 (0.43-0.73)	$2.8x10^{-5}$
1	rs2297901	*CACNA1S*	A	C	0.16	0.10	1.75 (1.34-2.28)	$4.0x10^{-5}$
5	rs889295	*ITGA1*	A	G	0.36	0.46	0.68 (0.56-0.82)	$5.2x10^{-5}$
5	rs889294	*ITGA1*	G	A	0.36	0.46	0.68 (0.56-0.82)	$5.2x10^{-5}$
4	rs66638185	*BANK1*	G	A	0.22	0.30	0.65 (0.53-0.81)	$6.7x10^{-5}$
14	rs61251127	*NFKBIA**	A	G	0.20	0.28	0.64 (0.51-0.80)	$7.2x10^{-5}$
5	rs13161948	*OR2Y1**	G	A	0.46	0.37	1.44 (1.20-1.73)	$8.5x10^{-5}$
14	rs9743508	*NFKBIA**	G	A	0.20	0.28	0.64 (0.51-0.80)	$8.9x10^{-5}$
14	rs8019505	*NFKBIA**	A	G	0.20	0.28	0.64 (0.51-0.80)	$9.5x10^{-5}$
14	rs17514338	*NFKBIA**	A	G	0.20	0.28	0.64 (0.51-0.80)	$9.5x10^{-5}$
11	rs12223477	*ACER3*	A	G	0.49	0.40	1.43 (1.20-1.71)	$9.7x10^{-5}$
4	rs4699259	*BANK1*	A	C	0.22	0.30	0.66 (0.54-0.81)	$9.8x10^{-5}$
4	rs4699261	*BANK1*	A	G	0.22	0.30	0.66 (0.54-0.81)	$9.9x10^{-5}$
4	rs34851381	*BANK1*	G	A	0.22	0.30	0.66 (0.54-0.81)	$9.9x10^{-5}$
4	rs17266357	*BANK1*	G	A	0.22	0.30	0.66 (0.54-0.81)	$9.9x10^{-5}$
4	rs35194352	*BANK1*	G	A	0.22	0.30	0.66 (0.54-0.81)	$9.9x10^{-5}$
4	rs13146194	*BANK1*	G	A	0.22	0.30	0.66 (0.54-0.81)	$9.9x10^{-5}$
4	rs11929782	*BANK1*	G	A	0.22	0.30	0.66 (0.54-0.81)	$9.9x10^{-5}$
4	rs10446708	*BANK1*	G	A	0.22	0.30	0.66 (0.54-0.81)	$9.9x10^{-5}$
11	rs3740766	*ACER3*	G	A	0.49	0.41	1.43 (1.19-1.71)	$1.0x10^{-4}$
5	rs11746643	*SPEF2*	G	A	0.34	0.43	0.69 (0.57-0.83)	$1.0x10^{-4}$
4	rs17200433	*BANK1*	G	A	0.22	0.30	0.66 (0.54-0.81)	$1.0x10^{-4}$
4	rs1500801	*PALLD*	A	T	0.25	0.19	1.55 (1.24-1.93)	$1.1x10^{-4}$
4	rs11940244	*BANK1*	A	C	0.22	0.30	0.66 (0.54-0.82)	$1.1x10^{-4}$
4	rs35838403	*BANK1*	A	G	0.22	0.30	0.66 (0.54-0.82)	$1.1x10^{-4}$
4	rs35416717	*BANK1*	A	C	0.22	0.30	0.66 (0.54-0.82)	$1.1x10^{-4}$
4	rs11931087	*BANK1*	G	A	0.22	0.30	0.66 (0.54-0.82)	$1.1x10^{-4}$
4	rs4699262	*BANK1*	A	G	0.22	0.30	0.66 (0.54-0.82)	$1.1x10^{-4}$
4	rs17266552	*BANK1*	A	G	0.22	0.30	0.66 (0.54-0.82)	$1.1x10^{-4}$
6	rs169858	*TAGAP**	A	G	0.30	0.22	1.49 (1.22-1.82)	$1.2x10^{-4}$
4	rs13117264	*BANK1*	C	A	0.23	0.30	0.67 (0.54-0.82)	$1.2x10^{-4}$
4	rs10446682	*BANK1*	A	G	0.22	0.30	0.67 (0.54-0.82)	$1.3x10^{-4}$
4	rs145580853	*BANK1*	A	T	0.22	0.29	0.66 (0.54-0.82)	$1.3x10^{-4}$
4	rs11935577	*BANK1*	A	C	0.23	0.30	0.67 (0.54-0.82)	$1.3x10^{-4}$
4	rs11944577	*BANK1*	G	A	0.23	0.30	0.67 (0.54-0.82)	$1.3x10^{-4}$
4	rs11944613	*BANK1*	G	A	0.23	0.30	0.67 (0.54-0.82)	$1.3x10^{-4}$
4	rs4496585	*BANK1*	G	A	0.23	0.30	0.67 (0.54-0.82)	$1.3x10^{-4}$
4	rs34499378	*BANK1*	A	G	0.22	0.30	0.67 (0.54-0.82)	$1.3x10^{-4}$
8	rs6469840	*ENPP2**	A	G	0.43	0.35	1.44 (1.19-1.73)	$1.3x10^{-4}$
3	rs2526388	*SEMA3F**	A	G	0.22	0.29	0.65 (0.52-0.81)	$1.4x10^{-4}$
6	rs212395	*TAGAP**	A	C	0.30	0.22	1.48 (1.21-1.81)	$1.4x10^{-4}$
6	rs212394	*TAGAP**	C	G	0.30	0.22	1.48 (1.21-1.81)	$1.4x10^{-4}$
4	rs17031641	*BANK1*	G	A	0.23	0.30	0.67 (0.54-0.82)	$1.5x10^{-4}$
5	rs13181141	*OR2Y1**	G	A	0.45	0.36	1.42 (1.19-1.71)	$1.5x10^{-4}$
4	rs4398527	*BANK1*	G	A	0.23	0.30	0.67 (0.54-0.82)	$1.5x10^{-4}$
5	rs10075764	*IL7R**	G	A	0.30	0.39	0.69 (0.56-0.83)	$1.5x10^{-4}$
5	rs12697352	*IL7R**	A	G	0.30	0.39	0.69 (0.57-0.84)	$1.6x10^{-4}$
5	rs1961220	*IL7R**	A	G	0.30	0.39	0.69 (0.57-0.84)	$1.9x10^{-4}$
4	rs34261083	*BANK1*	A	G	0.22	0.29	0.67 (0.54-0.83)	$1.9x10^{-4}$
20	rs62186949	*SIRPG*	G	A	0.09	0.15	0.58 (0.43-0.77)	$1.9x10^{-4}$
5	rs10472984	*IL7R**	C	G	0.30	0.38	0.69 (0.57-0.84)	$2.0x10^{-4}$
6	rs212393	*TAGAP**	G	A	0.30	0.22	1.47 (1.20-1.79)	$2.0x10^{-4}$
4	rs4572885	*BANK1*	T	A	0.29	0.36	0.69 (0.57-0.84)	$2.1x10^{-4}$
4	rs6833764	*BANK1*	G	A	0.33	0.41	0.70 (0.58-0.85)	$2.1x10^{-4}$
4	rs10031210	*BANK1*	A	G	0.33	0.41	0.70 (0.59-0.85)	$2.1x10^{-4}$

7	rs1167797	*HIP1*	G	A	0.31	0.38	0.69 (0.57-0.84)	2.1 x10^{-4}
20	rs2023565	*SIRPG**	A	G	0.09	0.15	0.58 (0.43-0.77)	2.2x10^{-4}
7	rs10808299	*CACNA2D1*	A	C	0.54	0.46	1.41 (1.18-1.70)	2.2x10^{-4}
5	rs4869426	*SPEF2**	G	A	0.34	0.43	0.70 (0.58-0.85)	2.3x10^{-4}
4	rs80241059	*BANK1*	G	A	0.33	0.41	0.71 (0.59-0.85)	2.4x10^{-4}
4	rs7691030	*BANK1*	G	A	0.33	0.41	0.71 (0.59-0.85)	2.4x10^{-4}
4	rs77613358	*BANK1*	G	A	0.33	0.41	0.71 (0.59-0.85)	2.4x10^{-4}
4	rs4522865	*BANK1*	G	A	0.33	0.41	0.71 (0.59-0.85)	2.4x10^{-4}
4	rs7690997	*BANK1*	G	C	0.33	0.41	0.71 (0.59-0.85)	2.4x10^{-4}
7	rs1167802	*HIP1*	C	A	0.31	0.38	0.70 (0.59-0.85)	2.4x10^{-4}
1	rs12141935	*N/A*	C	G	0.27	0.35	0.69 (0.56-0.84)	2.4x10^{-4}
5	rs13436926	*SPEF2**	A	G	0.34	0.43	0.71 (0.58-0.85)	2.6x10^{-4}
4	rs7656409	*BANK1*	A	G	0.33	0.41	0.71 (0.59-0.85)	2.7x10^{-4}
1	rs6675683	*SCYLP1BP1**	C	G	0.36	0.43	0.70 (0.58-0.85)	2.7x10^{-4}
7	rs1544461	*CACNA2D1*	A	G	0.54	0.45	1.41 (1.17-1.69)	2.7x10^{-4}
8	rs17368310	*PKHD1L1*	G	C	0.09	0.05	1.90 (1.34-2.69)	2.8x10^{-4}
7	rs2040976	*CACNA2D1*	A	G	0.54	0.45	1.41 (1.17-1.69)	2.9x10^{-4}
4	rs7656627	*BANK1*	A	G	0.33	0.41	0.71 (0.59-0.85)	2.9x10^{-4}
7	rs1167799	*HIP1*	G	A	0.31	0.38	0.70 (0.58-0.85)	3.0x10^{-4}
6	rs394581	*TAGAP**	G	A	0.29	0.21	1.46 (1.19-1.79)	3.2x10^{-4}
4	rs6856202	*BANK1*	G	A	0.35	0.43	0.71 (0.59-0.86)	3.1x10^{-4}
4	rs7699179	*BANK1*	A	G	0.34	0.41	0.71 (0.59-0.86)	3.1x10^{-4}
5	rs2408632	*GRAMD3**	G	A	0.34	0.27	1.43 (1.18-1.75)	3.4x10^{-4}
4	rs4699258	*BANK1**	A	G	0.22	0.29	0.69 (0.56-0.84)	3.6x10^{-4}
12	rs28999107	*LTBR**	A	C	0.40	0.47	0.71 (0.59-0.86)	3.7x10^{-4}
4	rs7683892	*BANK1*	A	G	0.32	0.40	0.71 (0.59-0.86)	4.3x10^{-4}
5	rs931555	*SPEF2*	A	G	0.30	0.39	0.71 (0.58-0.86)	4.4x10^{-4}
4	rs12508078	*N/A*	A	G	0.18	0.12	1.57 (1.22-2.02)	5.3x10^{-4}
6	rs7772702	*TRAF3IP2**	A	G	0.55	0.47	1.38 (1.15-1.65)	5.4x10^{-4}
6	rs59813857	*TRAF3IP2*	A	G	0.42	0.50	0.73 (0.61–0.87)	5.7x10^{-4}
20	rs6043388	*SIRPG*	A	G	0.10	0.15	0.61 (0.46-0.81)	5.9x10^{-4}
20	rs4599	*SDC4*	G	A	0.24	0.18	1.47 (1.18-1.84)	6.2x10^{-4}
8	rs13250503	*N/A*	C	A	0.29	0.23	1.43 (1.16-1.75)	6.4x10^{-4}
7	rs1167793	*HIP1*	G	A	0.19	0.24	0.67 (0.54-0.84)	6.4x10^{-4}
12	rs561712	*NOS1*	A	G	0.35	0.42	0.72 (0.60–0.87)	6.4x10^{-4}
4	rs11731260	*N/A*	A	G	0.12	0.08	1.70 (1.25-2.30)	6.4x10^{-4}
6	rs212391	*TAGAP**	G	A	0.30	0.23	1.42 (1.16-1.73)	6.9x10^{-4}
20	rs8060	*SDC4*	G	A	0.24	0.18	1.47 (1.18-1.83)	7.2x10^{-4}
21	rs66733675	*UBASH3A*	G	A	0.53	0.45	1.37 (1.14-1.64)	7.4x10^{-4}
6	rs380885	*TAGAP**	A	G	0.30	0.23	1.41 (1.16-1.73)	7.6x10^{-4}
7	rs3113138	*N/A*	A	G	0.09	0.13	0.59 (0.44-0.80)	7.6x10^{-4}
15	rs514743	*CHRNA5*	T	A	0.32	0.39	0.71 (0.58-0.87)	7.7x10^{-4}
5	rs35925399	*EBF1**	A	T	0.17	0.23	0.67 (0.53-0.85)	7.8 x10^{-4}
3	rs9841622	*CACNA2D3**	A	G	0.43	0.50	0.73 (0.61–0.88)	8.3 x10^{-4}
3	rs9835631	*CACNA2D3**	A	C	0.43	0.50	0.73 (0.61–0.88)	8.3 x10^{-4}
21	rs3827231	*UBASH3A*	G	C	0.39	0.47	0.73 (0.61–0.88)	8.4 x10^{-4}
6	rs115837294	*HCP5**	A	C	0.10	0.07	1.76 (1.26-2.45)	8.4 x10^{-4}
4	rs7665090	*MANBA**	A	G	0.45	0.52	0.73 (0.61–0.88)	8.8 x10^{-4}
5	rs41406347	*EBF1**	G	C	0.17	0.23	0.68 (0.54-0.85)	9.0 x10^{-4}
5	rs13165442	*EBF1**	G	C	0.17	0.23	0.68 (0.54-0.85)	9.1 x10^{-4}
14	rs35922526	*NFKBIA**	A	G	0.09	0.15	0.61 (0.45-0.82)	9.2 x10^{-4}
6	rs1883137	*TRAF3IP2*	G	A	0.53	0.46	1.35 (1.13-1.62)	9.2 x10^{-4}
5	rs61438813	*EBF1**	A	G	0.17	0.23	0.68 (0.54-0.85)	9.5 x10^{-4}
5	rs13184642	*EBF1**	A	G	0.17	0.23	0.68 (0.54-0.85)	9.5 x10^{-4}
5	rs34703275	*EBF1**	C	G	0.17	0.23	0.68 (0.54-0.85)	9.5 x10^{-4}
5	rs2161357	*EBF1**	G	A	0.17	0.23	0.68 (0.54-0.85)	9.5 x10^{-4}
5	rs2112634	*EBF1**	G	A	0.17	0.23	0.68 (0.54-0.85)	9.5 x10^{-4}
5	rs4921318	*EBF1**	G	A	0.17	0.23	0.68 (0.54-0.85)	9.5 x10^{-4}
5	rs4921321	*EBF1**	G	A	0.17	0.23	0.68 (0.54-0.85)	9.5 x10^{-4}
5	rs4921322	*EBF1**	A	C	0.17	0.23	0.68 (0.54-0.85)	9.5 x10^{-4}
5	rs35939651	*EBF1**	C	G	0.17	0.23	0.68 (0.54-0.85)	9.5 x10^{-4}

5	rs35993258	*EBF1**	G	A	0.17	0.23	0.68 (0.54-0.85)	9.5 x10^{-4}
5	rs13173967	*EBF1**	G	A	0.17	0.23	0.68 (0.54-0.85)	9.5 x10^{-4}
5	rs3962162	*EBF1**	A	G	0.17	0.23	0.68 (0.54-0.85)	9.5 x10^{-4}
5	rs35308932	*EBF1**	C	A	0.17	0.23	0.68 (0.54-0.85)	9.5 x10^{-4}
5	rs4921340	*EBF1**	G	A	0.17	0.23	0.68 (0.54-0.85)	9.5 x10^{-4}
5	rs55874544	*EBF1**	A	G	0.17	0.23	0.68 (0.54-0.85)	9.5 x10^{-4}
5	rs34314314	*EBF1**	A	G	0.17	0.23	0.68 (0.54-0.85)	9.5 x10^{-4}
5	rs34440003	*EBF1**	A	G	0.17	0.23	0.68 (0.54-0.85)	9.5 x10^{-4}
5	rs35675336	*EBF1**	G	A	0.17	0.23	0.68 (0.54-0.85)	9.5 x10^{-4}
5	rs13154096	*EBF1**	G	A	0.17	0.23	0.68 (0.54-0.85)	9.5 x10^{-4}
5	rs4921163	*EBF1**	A	G	0.17	0.23	0.68 (0.54-0.85)	9.5 x10^{-4}
5	rs4921164	*EBF1**	A	G	0.17	0.23	0.68 (0.54-0.85)	9.5 x10^{-4}
5	rs13171130	*EBF1**	G	A	0.17	0.23	0.68 (0.54-0.85)	9.5 x10^{-4}
5	rs11950065	*EBF1**	A	G	0.17	0.23	0.68 (0.54-0.85)	9.5 x10^{-4}
5	rs34417785	*EBF1**	G	A	0.17	0.23	0.68 (0.54-0.85)	9.5 x10^{-4}
5	rs1469061	*EBF1**	A	G	0.17	0.23	0.68 (0.54-0.85)	9.5 x10^{-4}
5	rs13166310	*EBF1**	C	A	0.17	0.23	0.68 (0.54-0.85)	9.5 x10^{-4}
5	rs13187731	*EBF1**	G	C	0.17	0.23	0.68 (0.54-0.85)	9.5 x10^{-4}
12	rs10861325	*ALDH1L2*	G	A	0.43	0.36	1.36 (1.13-1.63)	9.7 x10^{-4}
16	rs8045276	*LAT**	A	G	0.30	0.37	0.72 (0.59-0.88)	9.9 x10^{-4}

LN; Lupus nephritis, n=377, LN-negative, n=714, MAF; minor allele frequency

* Closest annotated gene. N/A: Located more than 50 kb from closest gene.

Supplementary table 2. Association analysis of patients with proliferative nephritis vs SLE without nephritis in discovery cohort

CHR	SNP	Gene	Minor allele	Major allele	MAF^{PN+}	MAF^{LN-}	OR (95% CI)	P
5	rs889294	*ITGA1*	G	A	0.34	0.46	0.59 (0.46-0.76)	5,9E-05
5	rs889295	*ITGA1*	A	G	0.34	0.46	0.59 (0.46-0.76)	5,9E-05
4	rs6856202	*BANK1*	G	A	0.31	0.43	0.60 (0.47-0.77)	7,6E-05
15	rs7176022	*LMAN1L*	A	C	0.37	0.26	1.68(1.29-2.18)	1,0E-04
4	rs11132321	*PDLIM3**	G	A	0.18	0.10	1.92 (1.38-2.67)	1,2E-04
4	rs66638185	*BANK1*	G	A	0.19	0.30	0.56 (0.42-0.75)	1,3E-04
1	rs12741486	*N/A*	G	A	0.15	0.08	2.04 (1.41-2.95)	1,4E-04
4	rs7656627	*BANK1*	A	G	0.29	0.41	0.61 (0.47-0.79)	1,6E-04
4	rs10031210	*BANK1*	A	G	0.29	0.41	0.61 (0.47-0.79)	1,6E-04
4	rs7690997	*BANK1*	G	C	0.30	0.41	0.61 (0.48-0.79)	1,7E-04
4	rs7691030	*BANK1*	G	A	0.30	0.41	0.61 (0.48-0.79)	1,7E-04
4	rs77613358	*BANK1*	G	A	0.30	0.41	0.61 (0.48-0.79)	1,7E-04
4	rs80241059	*BANK1*	G	A	0.30	0.41	0.61 (0.48-0.79)	1,7E-04
4	rs4522865	*BANK1*	G	A	0.30	0.41	0.61 (0.48-0.79)	1,7E-04
15	rs7162232	*LMAN1L*	G	A	0.37	0.26	1.64 (1.26-2.13)	1,9E-04
4	rs6833764	*BANK1*	G	A	0.29	0.41	0.62 (0.48-0.79)	2,0E-04
15	rs1378938	*LMAN1L**	A	G	0.36	0.26	1.64 (1.26-2.13)	2,1E-04
4	rs7699179	*BANK1*	A	G	0.30	0.41	0.62 (0.48-0.80)	2,1E-04
4	rs17266357	*BANK1*	G	A	0.20	0.30	0.58 (0.43-0.77)	2,1E-04
4	rs13146194	*BANK1*	G	A	0.20	0.30	0.58 (0.43-0.77)	2,1E-04
4	rs10446708	*BANK1*	G	A	0.20	0.30	0.58 (0.43-0.77)	2,1E-04
4	rs11929782	*BANK1*	G	A	0.20	0.30	0.58 (0.43-0.77)	2,1E-04
4	rs4699261	*BANK1*	A	G	0.20	0.30	0.58 (0.43-0.77)	2,1E-04
4	rs35194352	*BANK1*	G	A	0.20	0.30	0.58 (0.43-0.77)	2,1E-04
4	rs34851381	*BANK1*	G	A	0.20	0.30	0.58 (0.43-0.77)	2,1E-04
15	rs11634474	*LMAN1L*	C	G	0.37	0.26	1.64 (1.26-2.13)	2,2E-04
11	rs1901630	*N/A*	G	A	0.51	0.40	1.59 (1.23-2.03)	2,3E-04
4	rs34499378	*BANK1*	A	G	0.20	0.30	0.58 (0.43-0.77)	2,3E-04
4	rs7656409	*BANK1*	A	G	0.29	0.41	0.62 (0.48-0-80)	2,3E-04
4	rs34261083	*BANK1*	A	G	0.19	0.29	0.57 (0.42-0.77)	2,5E-04
20	rs311496	*GMEB2*	A	G	0.23	0.16	1.73 (1.29-2.33)	2,7E-04
20	rs311489	*GMEB2*	A	G	0.23	0.16	1.73 (1.29-2.33)	2,7E-04
1	rs11577369	*N/A*	A	G	0.14	0.08	1.97 (1.37-2.84)	2,8E-04
2	rs34366526	*N/A*	C	A	0.36	0.27	1.60 (1.24-2.05)	2,8E-04
4	rs7683892	*BANK1*	A	G	0.29	0.40	0.62 (0.48-0.80)	2,9E-04
1	rs67317828	*N/A*	C	A	0.14	0.08	1.95 (1.36-2.81)	2,9E-04
4	rs17200433	*BANK1*	G	A	0.20	0.30	0.58 (0.43-0.80)	2,9E-04
2	rs4432412	*N/A*	G	C	0.37	0.27	1.59 (1.24-2.04)	3,0E-04
2	rs11684463	*N/A*	T	A	0.37	0.27	1.59 (1.24-2.04)	3,0E-04
6	rs115350531	*NOTCH4**	G	A	0.31	0.22	1.66 (1.26-2.19)	3,0E-04
4	rs13117264	*BANK1*	C	A	0.20	0.30	0.59 (0.44-0.78)	3,0E-04
10	rs1499247	*N/A*	A	G	0.34	0.44	0.62 (0.48-0.81)	3,0E-04
4	rs4496585	*BANK1*	G	A	0.20	0.30	0.59 (0.44-0.78)	3,2E-04
4	rs11935577	*BANK1*	A	C	0.20	0.30	0.59 (0.44-0.78)	3,2E-04
4	rs11944577	*BANK1*	G	A	0.20	0.30	0.59 (0.44-0.78)	3,2E-04
4	rs11944613	*BANK1*	G	A	0.20	0.30	0.59 (0.44-0.78)	3,2E-04
4	rs4699259	*BANK1*	A	C	0.20	0.30	0.59 (0.44-0.78)	3,4E-04
4	rs4398527	*BANK1*	G	A	0.20	0.30	0.59 (0.44-0.79)	3,4E-04
1	rs12749297	*N/A*	G	A	0.14	0.08	1.94 (1.35-2.78)	3,4E-04
4	rs17031641	*BANK1*	G	A	0.20	0.30	0.59 (0.44-0.79)	3,5E-04
6	rs7772702	*TRAF3IP2**	A	G	0.58	0.47	1.55 (1.22-1.98)	3,5E-04
6	rs116225804	*MCCD1**	A	T	0.38	0.47	0.63 (0.49-0.81)	3,6E-04
1	rs7547030	*N/A*	G	A	0.15	0.09	1.89 (1.33-2.69)	3,6E-04
2	rs1044973	*TGOLN2*	G	A	0.41	0.51	0.64 (0.50-0.82)	3,7E-04
4	rs35838403	*BANK1*	A	G	0.20	0.30	0.59 (0.44-0.79)	3,7E-04
4	rs4699262	*BANK1*	A	G	0.20	0.30	0.59 (0.44-0.79)	3,7E-04
4	rs17266552	*BANK1*	A	G	0.20	0.30	0.59 (0.44-0.79)	3,7E-04
4	rs35416717	*BANK1*	A	C	0.20	0.30	0.59 (0.44-0.79)	3,7E-04

4	rs11940244	*BANK1*	A	C	0.20	0.30	0.59 (0.44-0.79)	3,7E-04
4	rs11931087	*BANK1*	G	A	0.20	0.30	0.59 (0.44-0.79)	3,7E-04
8	rs35162506	*N/A*	T	A	0.36	0.26	1.60 (1.23-2.07)	4,0E-04
1	rs12727172	*N/A*	A	C	0.14	0.08	1.92 (1.34-2.76)	4,0E-04
20	rs8124927	*C20ORF107**	C	A	0.36	0.27	1.60 (1.23-2.07)	4,0E-04
6	rs1888274	*FBXO9*	A	G	0.15	0.09	1.89 (1.33-2.68)	4,0E-04
4	rs145580853	*BANK1*	A	T	0.20	0.29	0.59 (0.44-0.79)	4,0E-04
16	rs8045276	*LAT**	A	G	0.28	0.37	0.62 (0.47-0.81)	4,1E-04
9	rs4743805	*N/A*	A	G	0.23	0.16	1.71 (1.27-2.30)	4,2E-04
1	rs36106538	*N/A*	G	A	0.15	0.08	1.89 (1.32-2.68)	4,2E-04
1	rs7538646	*N/A*	A	G	0.15	0.08	1.89 (1.32-2.68)	4,2E-04
6	rs116672850	*NOTCH4**	G	A	0.28	0.20	1.66 (1.25-2.20)	4,2E-04
3	rs35971655	*FLJ78302**	T	A	0.39	0.29	1.60 (1.23-2.07)	4,2E-04
6	rs13208357	*N/A*	A	G	0.08	0.15	0.46 (0.30-0.71)	4,3E-04
3	rs6788723	*FLJ78302**	C	G	0.39	0.29	1.60 (1.23-2.07)	4,3E-04
3	rs36030342	*FLJ78302**	G	C	0.39	0.29	1.60 (1.23-2.07)	4,3E-04
3	rs34148191	*FLJ78302**	A	G	0.39	0.29	1.60 (1.23-2.07)	4,3E-04
3	rs34193387	*FLJ78302**	G	A	0.39	0.29	1.60 (1.23-2.07)	4,3E-04
3	rs34505903	*FLJ78302**	C	A	0.39	0.29	1.60 (1.23-2.07)	4,3E-04
3	rs112196711	*FLJ78302**	G	A	0.39	0.29	1.60 (1.23-2.07)	4,3E-04
3	rs7626249	*FLJ78302**	A	T	0.39	0.29	1.60 (1.23-2.07)	4,3E-04
3	rs35675823	*FLJ78302**	G	C	0.39	0.29	1.60 (1.23-2.07)	4,3E-04
3	rs963045	*FLJ78302**	C	A	0.39	0.29	1.60 (1.23-2.07)	4,3E-04
3	rs2157057	*FLJ78302**	A	G	0.39	0.29	1.60 (1.23-2.07)	4,3E-04
3	rs62244840	*FLJ78302**	A	C	0.39	0.29	1.60 (1.23-2.07)	4,3E-04
3	rs35053103	*FLJ78302**	G	A	0.39	0.29	1.60 (1.23-2.07)	4,3E-04
3	rs62244846	*FLJ78302**	G	A	0.39	0.29	1.60 (1.23-2.07)	4,3E-04
1	rs7529431	*N/A*	C	G	0.15	0.08	1.88 (1.32-2.68)	4,3E-04
3	rs6441964	*FLJ78302**	G	A	0.39	0.29	1.59 (1.23-2.07)	4,6E-04
1	rs12727236	*N/A*	A	G	0.14	0.08	1.92 (1.33-2.76)	4,6E-04
16	rs8060345	*LAT**	C	G	0.29	0.38	0.62 (0.48-0.81)	4,6E-04
19	rs2392648	*NFIC**	A	G	0.14	0.07	1.96 (1.34-2.85)	4,6E-04
8	rs2445027	*FLJ43582**	C	A	0.20	0.13	1.77 (1.29-2.43)	4,7E-04
8	rs2979655	*MCPH1*	C	A	0.20	0.13	1.73 (1.27-2.36)	4,7E-04
22	rs9616812	*SHANK3**	A	G	0.41	0.50	0.64 (0.50-0.82)	4,8E-04
1	rs34864335	*N/A*	A	C	0.15	0.08	1.87 (1.32-2.66)	4,8E-04
14	rs4633605	*BAZ1A**	A	G	0.26	0.18	1.68 (1.26-2.25)	4,9E-04
4	rs10446682	*BANK1*	A	G	0.20	0.30	0.60 (0.45-0.80)	4,9E-04
6	rs116116733	*NOTCH4**	C	G	0.29	0.20	1.65 (1.24-2.18)	5,0E-04
3	rs6785981	*FLJ78302**	C	A	0.40	0.29	1.58 (1.22-2.05)	5,0E-04
3	rs7433566	*FLJ78302**	A	C	0.39	0.29	1.59 (1.22-2.07)	5,1E-04
14	rs9743508	*NFKBIA**	G	A	0.19	0.28	0.58 (0.42-0.79)	5,3E-04
10	rs4918900	*AFAP1L2*	A	G	0.29	0.21	1.61 (1.23-2.10)	5,3E-04
3	rs7374671	*CCR3**	A	G	0.38	0.28	1.58 (1.22-2.06)	5,4E-04
1	rs36086732	*N/A*	A	C	0.14	0.08	1.90 (1.32-2.73)	5,4E-04
3	rs11923720	*CCR3**	A	G	0.38	0.28	1.58 (1.22-2.06)	5,4E-04
1	rs7533697	*N/A*	A	G	0.14	0.08	1.90 (1.32-2.73)	5,4E-04
2	rs10460587	*TGOLN2**	G	A	0.40	0.50	0.65 (0.51-0.83)	5,5E-04
10	rs4748021	*N/A*	A	G	0.15	0.09	1.87 (1.31-2.67)	5,5E-04
4	rs4572885	*BANK1*	T	A	0.26	0.36	0.62 (0.48-0.81)	5,5E-04
19	rs4807451	*NFIC**	G	A	0.14	0.07	1.94 (1.33-2.82)	5,5E-04
1	rs6699619	*N/A*	A	T	0.15	0.08	1.86 (1.31-2.64)	5,5E-04
1	rs6687105	*N/A*	A	C	0.15	0.08	1.86 (1.31-2.64)	5,5E-04
22	rs9628185	*SHANK3**	G	A	0.41	0.50	0.64 (0.50-0.83)	5,6E-04
2	rs1053560	*TGOLN2*	G	A	0.41	0.51	0.65 (0.51-0.83)	5,7E-04
2	rs1061782	*TGOLN2*	G	A	0.41	0.51	0.65 (0.51-0.83)	5,7E-04
2	rs4832164	*TGOLN2*	A	C	0.41	0.51	0.65 (0.51-0.83)	5,7E-04
2	rs7607284	*TGOLN2*	C	G	0.41	0.51	0.65 (0.51-0.83)	5,7E-04
2	rs4459734	*TGOLN2*	C	A	0.41	0.51	0.65 (0.51-0.83)	5,7E-04
2	rs6547612	*TGOLN2*	G	A	0.41	0.51	0.65 (0.51-0.83)	5,7E-04
2	rs7572750	*TGOLN2*	C	A	0.41	0.51	0.65 (0.51-0.83)	5,7E-04

1	rs34749125	*N/A*	G	A	0.15	0.08	1.86 (1.31-2.64)	5,7E-04
2	rs7608881	*TGOLN2**	C	G	0.41	0.51	0.65 (0.51-0.83)	5,7E-04
1	rs4367796	*N/A*	A	G	0.15	0.08	1.86 (0.31-2.64)	5,7E-04
16	rs11648430	*LAT**	A	G	0.29	0.38	0.63 (0.48-0.82)	5,8E-04
14	rs17514338	*NFKBIA**	A	G	0.19	0.28	0.58 (0.43-0.79)	5,8E-04
14	rs8019505	*NFKBIA**	A	G	0.19	0.28	0.58 (0.43-0.79)	5,8E-04
14	rs61251127	*NFKBIA**	A	G	0.19	0.28	0.58 (0.43-0.79)	5,8E-04
2	rs10460586	*TGOLN2*	G	A	0.40	0.50	0.65 (0.51-0.83)	5,8E-04
3	rs9852052	*CCR3**	G	A	0.39	0.29	1.58 (1.22-2.05)	5,8E-04
3	rs11711054	*CCR3**	G	A	0.39	0.29	1.58 (1.22-2.05)	5,8E-04
3	rs7372094	*CCR3**	G	A	0.39	0.29	1.58 (1.22-2.05)	5,8E-04
3	rs6794300	*CCR3**	G	C	0.39	0.29	1.58 (1.22-2.05)	5,8E-04
3	rs9813756	*CCR3**	A	G	0.39	0.29	1.58 (1.22-2.05)	5,8E-04
3	rs9856098	*CCR3**	G	A	0.39	0.29	1.58 (1.22-2.05)	5,8E-04
3	rs11130089	*CCR3**	A	C	0.39	0.29	1.58 (1.22-2.05)	5,8E-04
3	rs10865940	*CCR3**	G	A	0.39	0.29	1.58 (1.22-2.05)	5,8E-04
3	rs6441961	*FLJ78302**	A	G	0.39	0.29	1.58 (1.22-2.05)	5,8E-04
3	rs6441962	*FLJ78302**	G	C	0.39	0.29	1.58 (1.22-2.05)	5,8E-04
3	rs6771900	*FLJ78302**	G	C	0.39	0.29	1.58 (1.22-2.05)	5,8E-04
3	rs6775208	*FLJ78302**	C	G	0.39	0.29	1.58 (1.22-2.05)	5,8E-04
18	rs17084788	*N/A*	G	A	0.19	0.12	1.75 (1.27-2.41)	5,9E-04
14	rs12433012	*NFKBIA**	A	G	0.10	0.18	0.51 (0.34-0.75)	6,1E-04
1	rs17409743	*DPH5**	G	A	0.14	0.08	1.89 (1.31-2.71)	6,1E-04
1	rs12741164	*N/A*	A	C	0.14	0.08	1.89 (1.31-2.71)	6,1E-04
1	rs34589233	*N/A*	A	G	0.14	0.08	1.89 (1.31-2.71)	6,1E-04
1	rs35918661	*N/A*	A	G	0.14	0.08	1.89 (1.31-2.71)	6,1E-04
1	rs7531958	*N/A*	A	G	0.14	0.08	1.89 (1.31-2.71)	6,1E-04
14	rs1568199	*BAZ1A**	A	G	0.26	0.18	1.66 (1.24-2.22)	6,1E-04
14	rs2383686	*BAZ1A**	T	A	0.26	0.18	1.66 (1.24-2.22)	6,1E-04
20	rs6135817	*N/A*	A	G	0.19	0.12	1.79 (1.28-2.51)	6,2E-04
3	rs6441963	*FLJ78302**	A	G	0.43	0.33	1.55 (1.21-2.00)	6,3E-04
6	rs1340064	*PRDM1**	G	C	0.55	0.44	1.54 (1.20-1.98)	6,5E-04
10	rs11004384	*PCDH15*	C	A	0.39	0.30	1.56 (1.21-2.01=	6,5E-04
10	rs4935057	*N/A*	A	G	0.45	0.35	1.53 (1.20-1.96)	6,7E-04
2	rs993648	*CERKL*	A	G	0.18	0.27	0.59 (0.43-0.80)	6,7E-04
14	rs56173145	*NFKBIA**	A	G	0.19	0.18	0.51 (0.35-0.75)	7,0E-04
1	rs35576543	*DPH5**	G	A	0.14	0.08	1.87 (1.30-2.69)	7,0E-04
1	rs10493943	*N/A*	G	A	0.14	0.08	1.87 (1.30-2.69)	7,0E-04
1	rs4409674	*N/A*	A	C	0.14	0.08	1.87 (1.30-2.69)	7,0E-04
6	rs1476044	*TRAF3IP2**	A	G	0.53	0.43	1.52 (1.20-1.95)	7,1E-04
1	rs71662918	*N/A*	A	G	0.15	0.09	1.84 (1.29-2.61)	7,2E-04
8	rs13250503	*N/A*	C	A	0.32	0.23	1.58 (1.21-2.05)	7,2E-04
9	rs10814139	*CCL21**	C	G	0.56	0.47	1.54 (1.20-1.98)	7,3E-04
1	rs17450565	*N/A*	G	A	0.15	0.08	1.84 (1.29-2.62)	7,4E-04
10	rs7073883	*PCDH15*	G	A	0.38	0.28	1.55 (1.20-2.00)	7,6E-04
20	rs6131030	*CDH22*	A	G	0.46	0.37	1.54 (1.20 (1.98)	7,8E-04
16	rs8062784	*A2BP1*	A	T	0.10	0.06	2.12 (1.37-3.28)	7,9E-04
3	rs2526388	*SEMA3F**	A	G	0.20	0.29	0.59 (0.43-0.80)	7,9E-04
6	rs1794818	*FYN**	A	G	0.36	0.45	0.65 (0.50-0.83)	8,0E-04
4	rs7663030	*SHROOM3**	A	G	0.07	0.13	0.46 (0.29-0.72)	8,0E-04
1	rs6675683	*SCYL1BP1**	C	G	0.34	0.43	0.65 (0.50.83)	8,2E-04
1	rs12120384	*N/A*	T	A	0.40	0.31	1.56 (1.20-2.02)	8,4E-04
5	rs4869426	*SPEF2**	G	A	0.32	0.43	0.64 (0.50-0.839	8,4E-04
16	rs11642176	*N/A*	A	C	0.24	0.17	1.63 (1.22-2.18)	8,5E-04
20	rs202476	*SIRPG**	A	G	0.34	0.43	0.64 (0.49-0.83)	8,6E-04
20	rs202473	*SIRPG**	A	T	0.34	0.43	0.64 (0.49-0.83)	8,6E-04
20	rs497134	*SIRPG**	A	G	0.34	0.43	0.64 (0.49-0.83)	8,6E-04
10	rs6602141	*RSU1*	G	A	0.35	0.45	0.65 (0.50-0.84)	8,7E-04
10	rs4935058	*N/A*	G	C	0.45	0.35	1.52 (1.19-1.94)	8,7E-04
10	rs10824915	*N/A*	G	A	0.45	0.35	1.52 (1.19-1.94)	8,7E-04
2	rs6709762	*ALK*	G	A	0.19	0.12	1.76 (1.26-2.45)	8,8E-04

17	rs7211524	*PRKCA*	A	G	0.09	0.04	2.29 (1.41-3.74)	8,8E-04
3	rs1799865	*FLJ78302**	G	A	0.40	0.30	1.55 (1.20 -2.00)	8,8E-04
3	rs3138042	*FLJ78302**	G	A	0.40	0.30	1.55 (1.20-2.00)	8,8E-04
10	rs10824911	*N/A*	A	T	0.45	0.35	1.52 (1.19-1.94)	8,8E-04
10	rs1962474	*N/A*	G	A	0.45	0.35	1.52 (1.19-1.94)	8,8E-04
10	rs1011516	*N/A*	G	A	0.45	0.35	1.52 (1.19-1.94)	8,8E-04
1	rs10916541	*N/A*	C	A	0.44	0.35	1.53 (1.19-1.97)	8,9E-04
5	rs13436926	*SPEF2**	A	G	0.32	0.43	0.65 (0.50-0.84)	8,9E-04
6	rs3777921	*TRAF3IP2*	G	A	0.56	0.46	1.50 (1.18-1.91)	8,9E-04
19	rs1205318	*LILRA4**	A	G	0.32	0.23	1.57 (1.20-2.05)	9,2E-04
10	rs4440941	*N/A*	A	G	0.45	0.35	1.51 (1.18-1.93)	9,3E-04
3	rs2097282	*FLJ78302**	G	A	0.40	0.30	1.54 (1.19-1.99)	9,4E-04
3	rs3918358	*FLJ78302**	C	A	0.40	0.30	1.54 (1.19-1.99)	9,4E-04
6	rs10872070	*TRAF3IP2*	G	A	0.56	0.46	1.50 (1.18-1.90)	9,5E-04
5	rs11746643	*SPEF2**	G	A	0.32	0.43	0.65 (0.50-0.84)	9,5E-04
2	rs10209375	*ALK*	C	G	0.18	0.11	1.76 (1.26-2.47)	9,6E-04
3	rs6441972	*FLJ78302**	A	G	0.40	0.30	1.54 (1.19-1.99)	9,8E-04
1	rs12746449	*N/A*	A	G	0.15	0.09	1.78 (1.27-2.55)	1,0E-03

Proliferative nephritis, n=173, LN-negative, n=714, MAF; minor allele frequency

* Closest annotated gene. N/A: Located more than 50 kb from closest gene.

Supplementary table 3. Association analysis of LN patients with ESRD vs SLE without nephritis in the discovery cohort

CHR	SNP	Gene	Minor allele	Major allele	MAF^{ESRD+}	MAF^{LN-}	OR	P
10	rs2763321	*MPP7*	A	G	0.46	0.22	3.22 (1.93-5.37)	7,4E-06
2	rs62180200	*N/A*	A	G	0.22	0.08	4.22 (2.21-8.04)	1,3E-05
2	rs7577144	*N/A*	A	G	0.22	0.08	4.12 (2.14-7.93)	2,2E-05
2	rs11904405	*N/A*	A	C	0.21	0.08	3.94 (2.06-7.53)	3,4E-05
2	rs6433896	*N/A*	A	G	0.21	0.08	3.94 (2.06-7.53)	3,4E-05
2	rs6738120	*N/A*	A	G	0.21	0.08	3.94 (2.06-7.53)	3,4E-05
2	rs6719497	*N/A*	C	A	0.21	0.08	3.94 (2.06-7.53)	3,4E-05
2	rs12373582	*LOC389073**	G	A	0.25	0.10	3.21 (1.80-5.74)	8,0E-05
8	rs10957293	*N/A*	A	C	0.14	0.05	4.24 (2.05-8.80)	1,0E-04
8	rs10957294	*N/A*	G	A	0.14	0.05	4.24 (2.05-8.80)	1,0E-04
10	rs12573804	*N/A*	A	G	0.14	0.04	4.77 (2.17-10.48)	1,0E-04
1	rs11581332	*IL23R**	C	G	0.14	0.05	4.54 (2.11-9.75)	1,1E-04
5	rs2431873	*ANKRD55*	G	A	0.26	0.11	3.05 (1.72-5.40)	1,3E-04
12	rs2284136	*KCNA5**	A	G	0.17	0.37	0.29 (0.15-0.55)	1,5E-04
4	rs10029121	*N/A*	C	G	0.29	0.13	2.92 (1.67-5.10)	1,7E-04
2	rs62180169	*N/A*	A	G	0.19	0.08	3.88 (1.91-7.88)	1,8E-04
6	rs4708114	*N/A*	A	G	0.57	0.35	2.61 (1.58-4.32)	1,8E-04
7	rs17150238	*HDAC9*	G	A	0.42	0.21	2.63 (1.58-4.36)	1,8E-04
4	rs870660	*MGC21874*	G	A	0.67	0.47	2.75 (1.62-4.66)	1,9E-04
5	rs4295390	*FBXL7*	G	A	0.50	0.32	2.57 (1.56-4.25)	2,2E-04
7	rs10235067	*HDAC9*	G	A	0.45	0.23	2.52 (1.54-4.11)	2,2E-04
18	rs79258392	*N/A*	A	C	0.17	0.06	3.51 (1.78-6.90)	2,8E-04
6	rs174375	*TRAF3IP2*	C	G	0.16	0.35	0.30 (0.16-0.57)	3,0E-04
6	rs174392	*TRAF3IP2*	C	A	0.12	0.32	0.26 (0.13-0.54)	3,1E-04
6	rs174393	*TRAF3IP2*	G	A	0.12	0.32	0.26 (0.13-0.54)	3,1E-04
14	rs857058	*N/A*	A	G	0.14	0.04	3.93 (1.86-8.28)	3,3E-04
6	rs174399	*TRAF3IP2*	G	A	0.12	0.32	0.26 (0.13-0.54)	3,3E-04
1	rs12125458	*PTGFRN**	A	C	0.24	0.10	2.94 (1.62-5.34)	3,8E-04
5	rs10053108	*FBXL7*	G	A	0.49	0.32	2.47 (1.50-4.07)	3,9E-04
4	rs33952102	*N/A*	D	I	0.28	0.13	2.76 (1.57-4.84)	4,0E-04
10	rs12776510	*ANK3**	G	A	0.41	0.25	2.63 (1.54-4.50)	4,0E-04
15	rs4411466	*N/A*	G	A	0.22	0.09	3.14 (1.67-5.91)	4,0E-04
1	rs10923819	*HSD3B2**	G	A	0.49	0.30	2.39 (1.48-3.88)	4,0E-04
7	rs13234273	*HDAC9*	A	G	0.22	0.09	3.07 (1.65-5.71)	4,1E-04
9	rs3125007	*NOTCH1*	G	A	0.17	0.39	0.33 (0.18-0.61)	4,1E-04
3	rs4234274	*ALDH1L1*	C	A	0.38	0.19	2.44 (1.49-4.00)	4,1E-04
12	rs2160086	*CD69*	A	T	0.29	0.49	0.37 (0.21-0.64)	4,2E-04
3	rs6799479	*N/A*	A	C	0.30	0.16	2.69 (1.55-4.65)	4,2E-04
14	rs857062	*N/A*	G	A	0.14	0.04	3.81 (1.81-8.02)	4,2E-04
14	rs857061	*N/A*	G	A	0.14	0.04	3.81 (1.81-8.02)	4,2E-04
14	rs1026425	*N/A*	A	G	0.14	0.04	3.81 (1.81-8.02)	4,2E-04
14	rs76745971	*N/A*	G	A	0.14	0.04	3.81 (1.81-8.02)	4,2E-04
14	rs857057	*N/A*	G	A	0.14	0.04	3.81 (1.81-8.02)	4,2E-04
14	rs860719	*N/A*	G	A	0.14	0.04	3.81 (1.81-8.02)	4,2E-04
9	rs3125009	*NOTCH1*	A	G	0.17	0.39	0.33 (0.18-0.61)	4,4E-04
1	rs12563908	*N/A*	A	G	0.34	0.19	2.55 (1.51-4.31)	4,5E-04
12	rs3759334	*LTBR**	A	G	0.39	0.21	2.48 (1.49-4.11)	4,5E-04
6	rs174382	*TRAF3IP2*	A	G	0.20	0.39	0.35 (0.20-0.63)	4,6E-04
14	rs2776508	*N/A*	A	G	0.14	0.04	3.77 (1.80-7.93)	4,6E-04
14	rs2776507	*N/A*	G	A	0.14	0.04	3.77 (1.80-7.93)	4,6E-04
2	rs1115910	*AFF3*	G	A	0.64	0.45	2.50 (1.50-4.17)	4,7E-04
2	rs12995420	*AFF3*	G	A	0.64	0.45	2.50 (1.50-4.17)	4,7E-04
1	rs3102463	*C1orf100*	A	G	0.64	0.44	2.53 (1.50-4.25)	4,8E-04

9	rs9411209	*NOTCH1*	A	G	0.17	0.39	0.33 (0.18-0.62)	4,8E-04
6	rs174397	*TRAF3IP2*	G	A	0.14	0.34	0.30 (0.16-0.59)	4,8E-04
2	rs1062143	*AFF3*	G	A	0.64	0.45	2.48 (1.49-4.14)	4,9E-04
2	rs11685341	*AFF3*	A	G	0.62	0.43	2.45 (1.48-4.06)	5,0E-04
10	rs7909961	*ANK3**	A	G	0.39	0.25	2.58 (1.51-4.39)	5,1E-04
6	rs114067228	*SFTPG**	A	G	0.17	0.07	3.48 (1.72-7.04)	5,1E-04
6	rs116184320	*SFTPG**	A	G	0.17	0.07	3.48 (1.72-7.04)	5,1E-04
4	rs9997710	*GABRB1*	A	G	0.63	0.43	2.42 (1.47-4.00)	5,1E-04
22	rs5768239	*N/A*	G	A	0.21	0.09	2.94 (1.60-5.41)	5,2E-04
2	rs17030946	*THADA*	A	C	0.20	0.08	3.12 (1.64-5.93)	5,2E-04
2	rs7605430	*AFF3*	G	C	0.64	0.45	2.47 (1.48-4.13)	5,3E-04
21	rs2824714	*PRSS7*	G	A	0.34	0.18	2.55 (1.50-4.34)	5,4E-04
6	rs7748820	*TRAF3IP2**	A	G	0.16	0.35	0.31 (0.16-0.60)	5,4E-04
6	rs11153305	*TRAF3IP2**	G	A	0.16	0.35	0.31 (0.16-0.60)	5,4E-04
2	rs7606704	*N/A*	C	A	0.17	0.06	3.33 (1.68-6.59)	5,5E-04
2	rs62180188	*N/A*	G	A	0.17	0.07	3.40 (1.70-6.82)	5,6E-04
5	rs461425	*N/A*	G	A	0.18	0.07	3.27 (1.66-6.44)	5,9E-04
2	rs12712063	*AFF3*	A	G	0.47	0.30	2.38 (1.45-3.90)	6,0E-04
2	rs74600494	*THADA*	A	G	0.20	0.08	3.10 (1.62-5.91)	6,1E-04
2	rs79271345	*THADA*	A	G	0.20	0.08	3.10 (1.62-5.91)	6,1E-04
2	rs7558817	*AFF3*	A	C	0.47	0.30	2.37 (1.45-3.89)	6,1E-04
2	rs6542905	*AFF3*	G	C	0.47	0.30	2.37 (1.45-3.89)	6,1E-04
2	rs7599083	*AFF3*	A	G	0.47	0.30	2.37 (1.45-3.89)	6,1E-04
2	rs11887597	*AFF3*	G	A	0.59	0.40	2.38 (1.45-3.91)	6,2E-04
2	rs77791126	*THADA*	A	G	0.20	0.08	3.05 (1.61-5.78)	6,2E-04
10	rs7087726	*ANK3**	C	G	0.39	0.25	2.55 (1.49-4.37)	6,2E-04
2	rs62180167	*N/A*	A	G	0.17	0.07	3.29 (1.66-6.52)	6,2E-04
2	rs62180190	*N/A*	A	C	0.17	0.07	3.29 (1.66-6.52)	6,2E-04
2	rs62180186	*N/A*	G	A	0.17	0.07	3.29 (1.66-6.51)	6,3E-04
2	rs75514072	*THADA*	C	G	0.20	0.08	3.08 (1.62-5.89)	6,3E-04
2	rs17030967	*THADA*	A	G	0.20	0.08	3.08 (1.62-5.88)	6,4E-04
6	rs6568689	*TRAF3IP2**	A	G	0.28	0.38	0.34 (0.19-0.63)	6,4E-04
2	rs62178859	*N/A*	G	A	0.17	0.07	3.29 (1.66-6.50)	6,4E-04
10	rs4948420	*N/A*	A	G	0.24	0.12	2.77 (1.54-4.98)	6,5E-04
10	rs3808942	*ANK3**	G	A	0.39	0.25	2.54 (1.49-4.33)	6,6E-04
1	rs1538987	*HSD3B2**	G	A	0.49	0.30	2.30 (1.42-3.71)	6,7E-04
2	rs138162260	*THADA*	G	A	0.20	0.08	3.03 (1.60-5.74)	6,7E-04
16	rs2361755	*N/A*	C	G	0.21	0.09	2.91 (1.57-5.37)	6,7E-04
2	rs7573500	*THADA*	A	G	0.20	0.08	3.03 (1.60-5.73)	6,8E-04
2	rs17039133	*THADA*	G	A	0.20	0.08	3.03 (1.60-5.73)	6,9E-04
2	rs13409687	*THADA*	G	A	0.20	0.08	3.03 (1.60-5.73)	6,9E-04
2	rs7561114	*THADA*	A	C	0.20	0.08	3.03 (1.60-5.73)	6,9E-04
2	rs17030889	*THADA*	T	A	0.20	0.08	3.03 (1.60-5.73)	6,9E-04
2	rs6731980	*THADA*	C	A	0.20	0.08	3.03 (1.60-5.73)	6,9E-04
2	rs6717636	*THADA*	G	C	0.20	0.08	3.03 (1.60-5.73)	6,9E-04
2	rs17030890	*THADA*	G	A	0.20	0.08	3.03 (1.60-5.73)	6,9E-04
2	rs6747229	*THADA*	A	T	0.20	0.08	3.03 (1.60-5.73)	6,9E-04
2	rs78497923	*THADA*	A	C	0.20	0.08	3.03 (1.60-5.73)	6,9E-04
2	rs9308833	*AFF3*	A	G	0.47	0.30	2.35 (1.43-3.84)	7,0E-04
10	rs1599166	*ANK3**	G	A	0.39	0.25	2.52 (1.48-4.30)	7,0E-04
2	rs74821293	*THADA*	G	A	0.20	0.08	3.02 (1.59-5.72)	7,0E-04
2	rs10189235	*THADA*	G	C	0.20	0.08	3.02 (1.59-5.72)	7,0E-04
2	rs76851696	*THADA*	G	A	0.20	0.08	3.02 (1.59-5.72)	7,0E-04
10	rs12411733	*ANK3*	A	G	0.47	0.31	2.35 (1.43-3.85)	7,0E-04
2	rs72865267	*THADA*	G	A	0.20	0.08	3.02 (1.59-5.72)	7,1E-04
10	rs10761495	*ANK3**	A	C	0.63	0.46	2.42 (1.45-4.03)	7,2E-04

2	rs17030925	*THADA*	C	A	0.20	0.08	3.01 (1.59-5.71)	7,2E-04
12	rs10772127	*CD69**	G	A	0.28	0.46	0.38 (0.22-0.67)	7,3E-04
2	rs75607314	*THADA*	A	C	0.20	0.08	3.01 (1.59-5.70)	7,4E-04
12	rs3176787	*CD69*	A	G	0.29	0.48	0.39 (0.22-0.67)	7,4E-04
12	rs3176773	*CD69**	G	A	0.29	0.48	0.39 (0.22-0.67)	7,4E-04
12	rs7309875	*CD69**	A	G	0.29	0.48	0.39 (0.22-0.67)	7,4E-04
6	rs174373	*TRAF3IP2*	A	G	0.07	0.25	0.20 (0.08-0.51)	7,4E-04
12	rs3176776	*CD69**	C	A	0.28	0.46	0.38 (0.22-0.67)	7,4E-04
12	rs3176775	*CD69**	A	T	0.28	0.46	0.38 (0.22-0.67)	7,4E-04
12	rs12427278	*CD69**	C	A	0.28	0.46	0.38 (0.22-0.67)	7,4E-04
12	rs12427301	*CD69**	G	A	0.28	0.46	0.38 (0.22-0.67)	7,4E-04
12	rs11052978	*CD69**	A	G	0.28	0.46	0.38 (0.22-0.67)	7,4E-04
12	rs10772128	*CD69**	A	G	0.28	0.46	0.38 (0.22-0.67)	7,4E-04
6	rs115032658	*DPCR1*	C	G	0.17	0.07	3.35 (1.66-6.76)	7,4E-04
10	rs1240375	*KIAA1975*	G	A	0.21	0.38	0.37 (0.20-0.66)	7,4E-04
2	rs6752448	*THADA*	C	G	0.20	0.08	3.01 (1.59-5.70)	7,4E-04
2	rs7572923	*THADA*	A	C	0.20	0.08	3.01 (1.59-5.70)	7,4E-04
2	rs7599799	*THADA*	G	A	0.20	0.08	3.01 (1.59-5.70)	7,4E-04
2	rs17030835	*THADA*	C	G	0.20	0.08	3.01 (1.59-5.70)	7,4E-04
2	rs79955955	*THADA*	T	A	0.20	0.08	3.01 (1.59-5.70)	7,4E-04
2	rs6746058	*THADA*	G	A	0.20	0.08	3.01 (1.59-5.70)	7,4E-04
2	rs17030845	*THADA*	A	G	0.20	0.08	3.01 (1.59-5.70)	7,4E-04
2	rs77377889	*THADA*	A	C	0.20	0.08	3.01 (1.59-5.70)	7,4E-04
2	rs7561287	*THADA*	G	A	0.20	0.08	3.01 (1.59-5.70)	7,4E-04
2	rs17030855	*THADA*	A	C	0.20	0.08	3.01 (1.59-5.70)	7,4E-04
2	rs17030859	*THADA*	A	G	0.20	0.08	3.01 (1.59-5.70)	7,4E-04
11	rs10767842	*MPPED2*	A	G	0.21	0.10	3.00 (1.59-5.68)	7,4E-04
2	rs13405076	*THADA*	G	A	0.20	0.08	3.00 (1.59-5.69)	7,5E-04
2	rs6544669	*THADA*	G	A	0.20	0.08	3.00 (1.59-5.69)	7,5E-04
2	rs1388387	*THADA*	G	A	0.20	0.08	3.00 (1.59-5.69)	7,5E-04
2	rs10180005	*THADA*	G	C	0.20	0.08	3.00 (1.59-5.69)	7,5E-04
2	rs13405776	*THADA*	A	G	0.20	0.08	3.00 (1.59-5.69)	7,5E-04
2	rs13419380	*THADA*	C	A	0.20	0.08	3.00 (1.59-5.69)	7,5E-04
12	rs7958251	*CD69**	A	G	0.29	0.48	0.39 (0.22-0.67)	7,5E-04
2	rs7564886	*THADA*	A	C	0.20	0.08	3.00 (1.58-5.69)	7,5E-04
10	rs7087958	*ANK3*	G	A	0.47	0.31	2.33 (1.43-3.82)	7,6E-04
2	rs6544666	*THADA*	A	G	0.20	0.08	3.00 (1.58-5.68)	7,6E-04
2	rs6708735	*THADA*	A	G	0.20	0.08	3.00 (1.58-5.68)	7,6E-04
4	rs4698773	*CCDC109B*	A	C	0.42	0.25	2.37 (1.43-3.92)	7,6E-04
10	rs10821753	*ANK3**	G	A	0.63	0.46	2.41 (1.44-4.02)	7,7E-04
2	rs6757251	*THADA*	A	G	0.20	0.08	2.99 (1.58-5.67)	7,7E-04
2	rs6544665	*THADA*	A	G	0.20	0.08	2.99 (1.58-5.67)	7,8E-04
12	rs12817268	*CD69**	A	G	0.28	0.46	0.38 (0.22-0.67)	7,9E-04
12	rs12819552	*CD69**	G	A	0.28	0.46	0.38 (0.22-0.67)	7,9E-04
12	rs4763716	*CLEC2D**	G	A	0.62	0.41	2.37 (1.43-3.92)	7,9E-04
12	rs7295897	*CD69**	T	A	0.28	0.46	0.38 (0.22-0.67)	8,0E-04
2	rs113047789	*UBE2E3**	T	A	0.17	0.07	3.20 (1.62-6.32)	8,0E-04
2	rs62178852	*N/A*	G	A	0.17	0.07	3.20 (1.62-6.32)	8,0E-04
2	rs961788	*AFF3*	G	A	0.63	0.45	2.40 (1.44-4.02)	8,0E-04
2	rs6544664	*THADA*	A	T	0.20	0.08	2.98 (1.57-5.66)	8,1E-04
2	rs75097983	*THADA*	G	A	0.20	0.08	2.98 (1.57-5.65)	8,1E-04
2	rs7590220	*THADA*	G	A	0.20	0.09	2.86 (1.55-5.29)	8,2E-04
2	rs10209110	*AFF3*	A	G	0.63	0.45	2.41 (1.44-4.03)	8,2E-04
2	rs76865040	*N/A*	C	A	0.18	0.08	3.21 (1.60-6.08)	8,3E-04
2	rs2309748	*AFF3*	T	A	0.47	0.30	2.32 (1.42-3.81)	8,3E-04
2	rs62180197	*N/A*	G	A	0.17	0.07	3.20 (1.62-6.32)	8,4E-04

12	rs3176774	*CD69**	A	C	0.46	0.28	2.37 (1.43-3.94)	8,5E-04
21	rs968063	*N/A*	A	C	0.17	0.07	3.29 (1.63-6.63)	8,6E-04
2	rs6714788	*AFF3*	A	G	0.63	0.45	2.39 (1.43-3.99)	8,7E-04
2	rs7582731	*AFF3*	G	A	0.63	0.45	2.39 (1.43-3.99)	8,7E-04
9	rs9411207	*NOTCH1*	A	G	0.21	0.42	0.38 (0.22-0.67)	8,8E-04
2	rs62180198	*N/A*	A	G	0.17	0.07	3.18 (1.61-6.29)	8,8E-04
2	rs77116456	*N/A*	C	A	0.17	0.07	3.18 (1.61-6.29)	8,8E-04
6	rs13191407	*MAP3K7**	C	A	0.46	0.27	2.25 (1.39 (3.62)	8,9E-04
4	rs10034143	*TAPT1**	A	G	0.17	0.07	2.97 (1.56-5.65)	9,2E-04
22	rs2267152	*NF2*	G	A	0.26	0.14	2.60 (1.48-4.58)	9,3E-04
10	rs12260709	*NKX2-3**	A	G	0.20	0.08	2.86 (1.53-5.33)	9,4E-04
6	rs1575335	*N/A*	G	A	0.55	0.35	2.26 (1.39-3.66)	9,5E-04
18	rs3786330	*SERPINB5*	C	A	0.25	0.13	2.56 (1.46-4.46)	9,6E-04
4	rs16893097	*TAPT1**	A	C	0.17	0.07	2.96 (1.55-5.63)	9,6E-04
11	rs11236787	*C11orf30**	A	G	0.11	0.28	0.27 (0.12-0.59)	9,8E-04
12	rs3759333	*LTBR**	A	G	0.39	0.22	2.31 (1.40-3.80)	9,8E-04
6	rs383917	*TRAF3IP2*	A	C	0.06	0.24	0.17 (0.06-0.49)	1,0E-03

ESRD; End-stage renal disease, n=38, LN-negative, n=714, MAF; minor allele frequency

* Closest annotated gene. N/A: Located more than 50 kb from closest gene.

D; deletion, I; insertion

Supplementary table 4. Association analysis of patients with proliferative nephritis vs SLE without nephritis in the discovery cohort and replication cohort 1 and meta-analysis.

					Discovery cohort				Replication cohort				Meta analysis
CHR	SNP	Gene*	Minor allele	Major allele	MAF^{PN+}	MAF^{LN-}	OR	P	MAF^{PN+}	MAF^{LN-}	OR	P	P
4	rs6856202	*BANK1*	G	A	0.31	0.43	0.60 (0.47-0.77)	7.6E-05	0.37	0.46	0.69 (0.47-1.00)	5.4E-02	1.3E-05
4	rs7656627	*BANK1*	A	G	0.29	0.41	0.61 (0.47-0.79)	1.6E-04	0.33	0.43	0.65 (0.44-0.97)	3.3E-02	1.5E-05
4	rs7699179	*BANK1*	A	G	0.30	0.41	0.62 (0.48-0.80)	2.1E-04	0.33	0.43	0.64 (0.43-0.95)	2.7E-02	1.6E-05
4	rs10031210	*BANK1*	A	G	0.29	0.41	0.61 (0.47-0.79)	1.6E-04	0.33	0.43	0.66 (0.45-0.99)	4.2E-02	1.9E-05
4	rs80241059	*BANK1*	G	A	0.30	0.41	0.61 (0.48-0.79)	1.7E-04	0.34	0.43	0.67 (0.45-0.98)	4.1E-02	2.0E-05
4	rs7691030	*BANK1*	G	A	0.30	0.41	0.61 (0.48-0.79)	1.7E-04	0.34	0.43	0.67 (0.45-0.99)	4.2E-02	2.0E-05
4	rs77613358	*BANK1*	G	A	0.30	0.41	0.61 (0.48-0.79)	1.7E-04	0.34	0.43	0.67 (0.45-0.99)	4.2E-02	2.0E-05
4	rs4522865	*BANK1*	G	A	0.30	0.41	0.61 (0.48-0.79)	1.7E-04	0.34	0.43	0.67 (0.45-0.99)	4.2E-02	2.0E-05
4	rs6833764	*BANK1*	G	A	0.29	0.41	0.62 (0.48-0.79)	2.0E-04	0.33	0.43	0.66 (0.45-0.98)	3.8E-02	2.2E-05
4	rs7656409	*BANK1*	A	G	0.29	0.41	0.62 (0.48-0.80)	2.3E-04	0.33	0.43	0.66 (0.45-0.98)	3.8E-02	2.5E-05
4	rs34499378	*BANK1*	A	G	0.20	0.30	0.58 (0.43-0.77)	2.3E-04	0.23	0.33	0.63 (0.41-0.98)	3.8E-02	2.5E-05
4	rs34851381	*BANK1*	G	A	0.20	0.30	0.58 (0.43-0.77)	2.1E-04	0.24	0.33	0.65 (0.42-0.99)	4.5E-02	2.8E-05
4	rs17266357	*BANK1*	G	A	0.20	0.30	0.58 (0.43-0.77)	2.1E-04	0.24	0.33	0.65 (0.42-0.99)	4.6E-02	2.8E-05
4	rs4699261	*BANK1*	A	G	0.20	0.30	0.58 (0.43-0.77)	2.1E-04	0.24	0.33	0.65 (0.42-0.99)	4.6E-02	2.8E-05
4	rs35194352	*BANK1*	G	A	0.20	0.30	0.58 (0.43-0.77)	2.1E-04	0.24	0.33	0.65 (0.42-0.99)	4.6E-02	2.8E-05
4	rs13146194	*BANK1*	G	A	0.20	0.30	0.58 (0.43-0.77)	2.1E-04	0.24	0.33	0.65 (0.42-0.99)	4.6E-02	2.8E-05
4	rs11929782	*BANK1*	G	A	0.20	0.30	0.58 (0.43-0.77)	2.1E-04	0.24	0.33	0.65 (0.42-0.99)	4.6E-02	2.8E-05
4	rs10446708	*BANK1*	G	A	0.20	0.30	0.58 (0.43-0.77)	2.1E-04	0.24	0.32	0.65 (0.42-0.99)	5.0E-02	3.1E-05
4	rs34261083	*BANK1*	A	G	0.19	0.29	0.57 (0.42-0.77)	2.5E-04	0.23	0.32	0.64 (0.41-0.99)	4.7E-02	3.4E-05
4	rs7683892	*BANK1*	A	G	0.29	0.40	0.62 (0.48-0.80)	2.9E-04	0.33	0.42	0.68 (0.46-1.00)	5.4E-02	4.3E-05
4	rs35838403	*BANK1*	A	G	0.20	0.30	0.59 (0.44-0.79)	3.7E-04	0.24	0.33	0.65 (0.42-1.00)	5.0E-02	5.2E-05
4	rs35416717	*BANK1*	A	C	0.20	0.30	0.59 (0.44-0.79)	3.7E-04	0.24	0.33	0.65 (0.43-1.00)	5.1E-02	5.3E-05
4	rs11931087	*BANK1*	G	A	0.20	0.30	0.59 (0.44-0.79)	3.7E-04	0.24	0.33	0.65 (0.43-1.00)	5.1E-02	5.3E-05
4	rs4699262	*BANK1*	A	G	0.20	0.30	0.59 (0.44-0.79)	3.7E-04	0.24	0.33	0.65 (0.43-1.00)	5.2E-02	5.4E-05
4	rs17266552	*BANK1*	A	G	0.20	0.30	0.59 (0.44-0.79)	3.7E-04	0.24	0.33	0.65 (0.43-1.00)	5.2E-02	5.4E-05
4	rs11935577	*BANK1*	A	C	0.20	0.30	0.59 (0.44-0.78)	3.2E-04	0.25	0.33	0.67 (0.44-1.00)	6.0E-02	5.5E-05
4	rs11944577	*BANK1*	G	A	0.20	0.30	0.59 (0.44-0.78)	3.2E-04	0.25	0.33	0.67 (0.44-1.00)	6.0E-02	5.5E-05
4	rs11944613	*BANK1*	G	A	0.20	0.30	0.59 (0.44-0.78)	3.2E-04	0.25	0.33	0.67 (0.44-1.00)	6.0E-02	5.5E-05
4	rs11940244	*BANK1*	A	C	0.20	0.30	0.59 (0.44-0.79)	3.7E-04	0.24	0.33	0.65 (0.43-1.00)	5.3E-02	5.6E-05
4	rs4496585	*BANK1*	G	A	0.20	0.30	0.59 (0.44-0.78)	3.2E-04	0.25	0.33	0.67 (0.44-1.00)	6.1E-02	5.6E-05
4	rs13117264	*BANK1*	C	A	0.20	0.30	0.59 (0.44-0.78)	3.0E-04	0.25	0.33	0.67 (0.44-1.00)	6.4E-02	5.7E-05
4	rs4699259	*BANK1*	A	C	0.20	0.30	0.59 (0.44-0.78)	3.4E-04	0.24	0.32	0.66 (0.43-1.00)	5.9E-02	5.7E-05
4	rs4398527	*BANK1*	G	A	0.20	0.30	0.59 (0.44-0.79)	3.4E-04	0.25	0.33	0.67 (0.44-1.00)	6.1E-02	6.0E-05
4	rs17031641	*BANK1*	G	A	0.20	0.30	0.59 (0.44-0.79)	3.5E-04	0.25	0.33	0.67 (0.44-1.00)	6.0E-02	6.0E-05
4	rs10446682	*BANK1*	A	G	0.20	0.30	0.60 (0.45-0.80)	4.9E-04	0.24	0.33	0.65 (0.43-1.00)	4.9E-02	6.7E-05
4	rs17200433	*BANK1*	G	A	0.20	0.30	0.58 (0.43-0.78)	2.9E-04	0.23	0.30	0.64 (0.41-1.06)	8.7E-02	7.0E-05
4	rs66638185	*BANK1*	G	A	0.19	0.30	0.56 (0.42-0.75)	1.3E-04	0.25	0.32	0.74 (0.46-1.18)	2.0E-02	8.7E-05
10	rs4935057	*N/A*	A	G	0.45	0.35	1.53 (1.20-1.96)	6.7E-04	0.48	0.39	1.42 (0.98-2.06)	6.8E-02	1.2E-04
2	rs10209375	*ALK*	C	G	0.18	0.11	1.76 (1.26-2.47)	9.6E-04	0.19	0.12	1.60 (0.99-2.58)	5.3E-02	1.4E-04
10	rs4440941	*N/A*	A	G	0.45	0.35	1.51 (1.18-1.93)	9.3E-04	0.48	0.39	1.41 (0.98-2.05)	6.7E-02	1.6E-04
8	rs35162506	*N/A*	T	A	0.36	0.26	1.60 (1.23-2.07)	4.0E-04	0.37	0.26	2.03 (0.73-5.61)	1.7E-02	1.6E-04
10	rs1962474	*N/A*	G	A	0.45	0.35	1.52 (1.19-1.94)	8.8E-04	0.48	0.39	1.41 (0.97-2.05)	7.2E-02	1.6E-04
10	rs1011516	*N/A*	G	A	0.45	0.35	1.52 (1.19-1.94)	8.8E-04	0.48	0.39	1.41 (0.97-2.05)	7.2E-02	1.6E-04
10	rs11004384	*PCDH15*	C	A	0.39	0.30	1.56 (1.21-2.01)	6.5E-04	0.33	0.27	1.37 (0.92-2.03)	1.2E-02	2.2E-04
10	rs10824915	*N/A*	G	A	0.45	0.35	1.52 (1.19-1.94)	8.7E-04	0.47	0.39	1.37 (0.95-1.99)	1.0E-01	2.3E-04
20	rs6135817	*N/A*	A	G	0.19	0.12	1.79 (1.28-2.51)	6.2E-04	0.17	0.13	1.43 (0.86-2.36)	1.7E-01	3.0E-04
10	rs7073883	*PCDH15*	G	A	0.38	0.28	1.55 (1.20-2.00)	7.6E-05	0.32	0.26	1.35 (0.90-2.02)	1.5E-01	3.0E-04
14	rs56173145	*NFKBIA**	A	G	0.10	0.18	0.51 (0.35-0.75)	7.0E-04	0.10	0.14	0.67 (0.35-1.27)	2.2E-01	4.0E-04
1	rs10916541	*N/A*	C	A	0.44	0.35	1.53 (1.19-1.97)	8.9E-04	0.42	0.36	1.28 (0.88-1.86)	1.9E-01	4.9E-04
14	rs61251127	*NFKBIA**	A	G	0.19	0.28	0.58 (0.43-0.79)	5.8E-04	0.21	0.25	0.77 (0.50-1.22)	2.6E-01	9.9E-04

Discovery cohort: proliferative nephritis, n=173, LN-negative, n=714, Replication cohort 1: proliferative nephritis, n=65, LN-negative, n=746.

MAF; minor allele frequency

* Closest annotated gene. N/A: Located more than 50KB from closest gene.

Supplementary table 5. Association analysis of ESRD vs SLE without nephritis in the discovery cohort and replication cohort 1 and meta-analysis

			Minor	Major	Discovery cohort				Replication cohort				Meta analysis
CHR	SNP	Gene	allele	allele	MAF^{ESRD+}	MAF^{LN-}	OR	P	MAF^{ESRD+}	MAF^{LN-}	OR	P	P
10	rs12573804	*N/A*	A	G	0.14	0.04	4.77 (2.17-10.48)	1.0E-04	0.07	0.04	2.88 (1.16-7.15)	2.3E-02	9.6E-06
22	rs2267152	*NF2*	G	A	0.26	0.14	2.60 (1.48-4.58)	9.3E-04	0.23	0.18	1.76 (1.04-2.97)	3.5E-02	1.4E-04
14	rs857062	*N/A*	G	A	0.14	0.04	3.81 (1.81-8.02)	4.2E-04	0.10	0.06	2.06 (0.99-4.28)	5.4E-02	9.0E-04
14	rs2776508	*N/A*	A	G	0.14	0.04	3.77 (1.80-7.93)	4.6E-04	0.10	0.06	2.04 (0.98-4.23)	5.7E-02	9.7E-04

ESRD; End-stage renal disease, MAF; minor allele frequency

Discovery cohort: ESRD, n=38, LN-negative, n=714, Replication cohort 1: ESRD, n=48, LN-negative, n=746.

* Closest annotated gene. N/A: Located more than 50KB from closest gene.

Supplementary table 6. Results from association analysis of patients with LN (n=354) vs SLE without nephritis (n=479) in replication cohort 2

CHR	SNP	Gene	Minor allele	Major allele	MAF^{LN+}	MAF^{LN-}	OR (95% CI)	P
4	rs4699259	*BANK1*	A	C	0.23	0.28	0.80 (0.64–1.0)	0.052
4	rs66638185	*BANK1*	C	T	0.25	0.29	0.85 (0.69-1.01)	0.14
1	rs2297901	*CACNA1S*	A	C	0.11	0.10	1.1 (0.81-1.55)	0.40
4	rs1500801	*PALLD*	A	T	0.2	0.24	1.0 (0.83-1.31)	0.72
8	rs17368310	*PKHD1L1*	C	G	0.05	0.05	0.90 (0.57-1.43)	0.65
14	rs12433012	*NFKB1A**	A	G	0.16	0.14	1.1 (0.86–1.5)	0.36
8	rs6469840	*ENPP2**	T	C	0.35	0.37	0.93 (0.76–1.1)	0.48
6	rs169858	*LOC105378083*	A	G	0.26	0.26	0.96 (0.76–1.2)	0.76
5	rs889295	*ITGA1*	A	G	0.44	0.43	1.0 (0.85–1.2)	0.78
5	rs889294	*ITGA1*	G	A	0.44	0.43	1.0 (0.85–1.2)	0.78

LN; Lupus nephritis, n=377, LN-negative, n=714, MAF; minor allele frequency

* Closest annotated gene. N/A: Located more than 50 kb from closest gene.

Supplementary Table 7. Genetic regulation of DNA methylation by lupus nephritis associated variants, meQTL analysis.

Gene	Chr	CpG site	SNP	Minor allele	Major allele	Slope	meQTL p-value
ITGAM	16	cg02846316	rs55742763	A	G	0.020	1.3E-15
ITGAM	16	cg02846316	rs11641202	G	A	0.019	3.3E-15
ITGAM	16	cg02846316	rs2359661	G	A	0.018	5.0E-13
FCGR3A	1	cg04384208	rs426615	C	A	-0.036	1.5E-12
ITGAM	16	cg02846316	rs7206295	C	T	-0.018	1.9E-09
ITGAM	16	cg02846316	rs4077810	C	T	-0.018	1.9E-09
BLK	8	cg21175976	rs10098782	C	T	0.057	2.3E-09
ITGAM	16	cg02846316	rs4594268	C	T	-0.018	2.4E-09
ITGAM	16	cg02846316	rs3087796	A	G	-0.018	2.4E-09
ITGAM	16	cg02846316	rs4597342	C	T	-0.018	2.4E-09
BLK	8	cg21175976	rs11250148	C	T	0.055	3.8E-09
BLK	8	cg21175976	rs13262953	A	G	0.055	3.8E-09
BLK	8	cg21175976	rs7834638	C	T	0.055	3.8E-09
BLK	8	cg21175976	rs10097015	T	C	0.055	3.8E-09
BLK	8	cg21175976	rs1042689	T	C	0.055	3.8E-09
BLK	8	cg21175976	rs2898289	A	G	0.055	4.1E-09
BLK	8	cg21175976	rs4841561	T	C	0.055	4.1E-09
BLK	8	cg21175976	rs13248757	C	T	0.061	5.5E-09
BLK	8	cg21175976	rs10098664	C	T	0.058	1.7E-08
ITGAM	16	cg15817542	rs4594268	C	T	0.034	4.0E-08
ITGAM	16	cg15817542	rs3087796	A	G	0.034	4.0E-08
ITGAM	16	cg15817542	rs4597342	C	T	0.034	4.0E-08
Intergenic	8	cg12840336	rs13248757	C	T	-0.028	5.1E-08
ITGAM	16	cg15817542	rs7206295	C	T	0.034	6.4E-08
ITGAM	16	cg15817542	rs4077810	C	T	0.034	6.4E-08
ITGAM	16	cg02846316	rs8048583	C	T	-0.016	7.8E-08
Intergenic	8	cg12840336	rs10098782	C	T	-0.025	1.0E-07
ITGAM	16	cg15817542	rs34550882	T	C	-0.045	1.0E-07
Intergenic	8	cg12840336	rs11250148	C	T	-0.025	1.1E-07
Intergenic	8	cg12840336	rs13262953	A	G	-0.025	1.1E-07
Intergenic	8	cg12840336	rs7834638	C	T	-0.025	1.1E-07
Intergenic	8	cg12840336	rs10097015	T	C	-0.025	1.1E-07
Intergenic	8	cg12840336	rs1042689	T	C	-0.025	1.1E-07
ITGAM	16	cg02846316	rs7499192	C	T	-0.016	1.1E-07
ITGAM	16	cg02846316	rs3815801	T	C	-0.016	1.1E-07
BLK	8	cg21175976	rs2279169	A	G	0.053	1.4E-07
ITGAM	16	cg15817542	rs3815801	T	C	0.032	1.6E-07
Intergenic	8	cg12840336	rs2898289	A	G	-0.024	2.2E-07
Intergenic	8	cg12840336	rs4841561	T	C	-0.024	2.2E-07
IRF5	7	cg12816198	rs3778753	A	G	-0.047	3.0E-07
ITGAM	16	cg15817542	rs1143683	T	C	-0.037	3.9E-07
ITGAM	16	cg15817542	rs7193268	T	C	-0.037	3.9E-07
ITGAM	16	cg15817542	rs1143678	T	C	-0.037	3.9E-07
ITGAM	16	cg15817542	rs9933520	G	A	-0.037	3.9E-07
PYCARD	16	cg07461837	rs11865830	A	G	0.024	4.3E-07
PYCARD	16	cg07461837	rs3764327	C	T	0.024	4.3E-07
PYCARD	16	cg07461837	rs7196256	A	T	0.024	4.3E-07
ITGAM	16	cg15817542	rs41476751	C	T	-0.038	4.4E-07
ITGAM	16	cg15817542	rs11865830	A	G	0.032	4.6E-07

ITGAM	16	cg15817542	rs3764327	C	T	0.032	4.6E-07
ITGAM	16	cg15817542	rs7196256	A	T	0.032	4.6E-07
Intergenic	8	cg12840336	rs2279169	A	G	-0.026	5.3E-07
Intergenic	16	cg03575939	rs4594268	C	T	-0.022	5.9E-07
Intergenic	16	cg03575939	rs3087796	A	G	-0.022	5.9E-07
Intergenic	16	cg03575939	rs4597342	C	T	-0.022	5.9E-07
ITGAM	16	cg15817542	rs7499192	C	T	0.030	6.2E-07
IRF5	7	cg12816198	rs3807307	T	C	-0.045	6.7E-07
ITGAM	16	cg02846316	rs11865830	A	G	-0.016	7.2E-07
ITGAM	16	cg02846316	rs3764327	C	T	-0.016	7.2E-07
ITGAM	16	cg02846316	rs7196256	A	T	-0.016	7.2E-07
Intergenic	16	cg03575939	rs7206295	C	T	-0.021	8.0E-07
Intergenic	16	cg03575939	rs4077810	C	T	-0.021	8.0E-07
BLK	8	cg21175976	rs11250146	C	G	-0.046	8.6E-07
PYCARD	16	cg07461837	rs8048583	C	T	0.022	1.1E-06
ITGAM	16	cg15817542	rs8048583	C	T	0.030	1.1E-06
PYCARD	16	cg07461837	rs3815801	T	C	0.023	1.4E-06
RBM5	3	cg05623727	rs2526388	C	T	0.020	1.6E-06
Intergenic	16	cg03575939	rs7499192	C	T	-0.020	1.8E-06
Intergenic	16	cg03575939	rs3815801	T	C	-0.020	1.8E-06
Intergenic	16	cg03575939	rs8048583	C	T	-0.020	1.9E-06
ITGAM	16	cg02846316	rs11150608	A	G	0.014	2.0E-06
ITGAM	16	cg02846316	rs7192161	T	C	0.014	2.2E-06
ITGAM	16	cg02846316	rs11150610	A	C	0.014	2.2E-06
IRF5	7	cg12816198	rs3807306	G	T	-0.044	2.3E-06
Intergenic	5	cg12125772	rs35925399	T	A	0.043	2.4E-06
ITGAM	16	cg02846316	rs11645653	C	T	0.016	3.2E-06
BLK	8	cg21175976	rs4841557	G	A	0.044	3.5E-06
BLK	8	cg21175976	rs7011778	C	T	0.044	3.5E-06
BLK	8	cg21175976	rs6988443	T	C	0.044	3.5E-06
GPX3	5	cg26891370	rs2233311	A	C	-0.047	3.6E-06
GPX3	5	cg26891370	rs72790107	T	C	-0.047	3.6E-06
GPX3	5	cg26891370	rs2233302	G	C	-0.047	3.6E-06
GPX3	5	cg26891370	rs72790109	A	G	-0.047	3.6E-06
GPX3	5	cg26891370	rs72790110	A	G	-0.047	3.6E-06
GPX3	5	cg26891370	rs72790117	A	G	-0.047	3.6E-06
IRF5	7	cg26616347	rs3807307	T	C	0.012	3.7E-06
BLK	8	cg21175976	rs11785333	A	G	0.044	4.1E-06
IRF5	7	cg12816198	rs7808907	T	C	-0.045	4.5E-06
SLC38A3	3	cg15335139	rs2526388	C	T	0.057	4.8E-06
PYCARD	16	cg07461837	rs7499192	C	T	0.021	4.8E-06
GPX3	5	cg26891370	rs72790116	C	G	-0.047	5.0E-06
BLK	8	cg21175976	rs4841558	T	C	0.044	5.5E-06
BLK	8	cg21175976	rs7007439	A	T	0.044	5.5E-06
IRF5	7	cg26616347	rs752637	T	C	0.012	5.6E-06
Intergenic	14	cg03549618	rs35922526	A	G	0.070	6.4E-06
IRF5	7	cg12816198	rs11761199	G	A	-0.042	1.0E-05
Intergenic	16	cg03575939	rs8061729	C	T	0.020	1.1E-05
Intergenic	16	cg03575939	rs41477949	T	A	0.020	1.1E-05
BLK	8	cg21175976	rs1042701	A	G	-0.045	1.4E-05
Intergenic	16	cg03575939	rs9937837	G	T	0.019	1.4E-05
Intergenic	16	cg03575939	rs7499077	A	G	0.019	1.4E-05
Intergenic	16	cg03575939	rs34594027	C	T	0.019	1.4E-05

Intergenic	5	cg18043514	rs35925399	T	A	0.048	1.6E-05
BLK	8	cg21175976	rs36048422	C	T	-0.045	1.6E-05
BLK	8	cg21175976	rs11783065	G	A	-0.045	1.6E-05
ITGAX	16	cg01486260	rs7188189	C	T	0.047	1.7E-05
Intergenic	16	cg03575939	rs9925985	C	A	0.019	1.7E-05
IRF5	7	cg26616347	rs3778753	A	G	0.011	1.7E-05
GPX3	5	cg26891370	rs56003867	C	T	-0.074	1.9E-05
Intergenic	5	cg08777316	rs35925399	T	A	0.076	2.4E-05
BLK	8	cg21175976	rs6983727	T	C	-0.044	2.4E-05
Intergenic	5	cg17032564	rs35925399	T	A	0.064	2.6E-05
ITGAM	16	cg02846316	rs28372932	A	G	0.013	2.7E-05
BLK	8	cg21175976	rs2729940	A	G	0.047	2.7E-05
ITGAM	16	cg15817542	rs34572943	A	G	-0.036	2.7E-05
ITGAM	16	cg02846316	rs41440449	G	A	0.013	2.8E-05
IRF5	7	cg12816198	rs10954213	G	A	-0.041	3.1E-05
Intergenic	16	cg03575939	rs11865830	A	G	-0.018	3.2E-05
Intergenic	16	cg03575939	rs3764327	C	T	-0.018	3.2E-05
Intergenic	16	cg03575939	rs7196256	A	T	-0.018	3.2E-05
Intergenic	14	cg03549618	rs12433012	A	G	0.063	4.1E-05
IRF5	7	cg26616347	rs7808907	T	C	0.011	4.2E-05
GPX3	5	cg26891370	rs9324672	A	C	-0.044	4.3E-05
Intergenic	14	cg03549618	rs56173145	A	G	0.063	4.6E-05
ITGAM	16	cg15817542	rs11861161	C	T	-0.035	4.7E-05
ITGAM	16	cg15817542	rs34082782	A	G	-0.034	5.0E-05
ITGAM	16	cg15817542	rs35314490	A	G	-0.034	5.0E-05
ITGAM	16	cg15817542	rs35472514	G	C	-0.034	5.0E-05
ITGAM	16	cg15817542	rs9936831	T	A	-0.034	5.0E-05
ITGAM	16	cg15817542	rs12917874	C	T	-0.034	5.0E-05
ITGAM	16	cg15817542	rs13338069	G	A	-0.034	5.0E-05
ITGAM	16	cg15817542	rs7190018	A	G	-0.034	5.0E-05
ITGAM	16	cg15817542	rs7190807	C	G	-0.034	5.0E-05
ITGAM	16	cg15817542	rs9888879	C	T	-0.034	5.0E-05
ITGAM	16	cg15817542	rs12928810	A	G	-0.034	5.0E-05
ITGAM	16	cg15817542	rs9888739	T	C	-0.034	5.0E-05
ITGAM	16	cg15817542	rs36001237	T	A	-0.034	5.0E-05
ITGAM	16	cg15817542	rs9745421	A	G	-0.034	5.0E-05
ITGAM	16	cg15817542	rs6565227	T	A	-0.034	5.0E-05
ITGAM	16	cg15817542	rs34767000	C	A	-0.034	5.0E-05
ITGAM	16	cg02846316	rs1143682	A	G	0.013	5.3E-05
ITGAM	16	cg15817542	rs9939679	C	A	-0.034	5.9E-05
IRF5	7	cg26616347	rs10954214	C	T	0.011	7.0E-05
ITGAM	16	cg15817542	rs1143679	A	G	-0.034	7.3E-05
ITGAX	16	cg01486260	rs4594268	C	T	0.024	7.3E-05
ITGAX	16	cg01486260	rs3087796	A	G	0.024	7.3E-05
ITGAX	16	cg01486260	rs4597342	C	T	0.024	7.3E-05
FAM167A	8	cg15137355	rs2729940	A	G	0.013	7.5E-05
PYDC1	16	cg16556397	rs9925985	C	A	0.022	8.2E-05
Intergenic	8	cg12840336	rs4841557	G	A	-0.019	8.6E-05
Intergenic	8	cg12840336	rs7011778	C	T	-0.019	8.6E-05
Intergenic	8	cg12840336	rs6988443	T	C	-0.019	8.6E-05
Intergenic	8	cg12840336	rs11785333	A	G	-0.018	9.1E-05
ITGAM	16	cg09743950	rs41476751	C	T	-0.030	9.6E-05
ITGAM	16	cg15817542	rs8061729	C	T	-0.027	9.7E-05

ITGAM	16	cg15817542	rs41477949	T	A	-0.027	9.7E-05
FCGR3A	1	cg04384208	rs1801274	A	G	0.027	9.7E-05
ITGAX	16	cg01486260	rs7192161	T	C	-0.022	9.9E-05
ITGAX	16	cg01486260	rs11150610	A	C	-0.022	9.9E-05
ITGAM	16	cg09743950	rs1143683	T	C	-0.029	9.9E-05
ITGAM	16	cg09743950	rs7193268	T	C	-0.029	9.9E-05
ITGAM	16	cg09743950	rs1143678	T	C	-0.029	9.9E-05
ITGAM	16	cg09743950	rs9933520	G	A	-0.029	9.9E-05
IRF5	7	cg26616347	rs3807306	G	T	0.010	9.9E-05
ITGAM	16	cg09743950	rs34550882	T	C	-0.033	1.3E-04
ITGAX	16	cg01486260	rs7206295	C	T	0.023	1.4E-04
ITGAX	16	cg01486260	rs4077810	C	T	0.023	1.4E-04
ITGAM	16	cg15817542	rs9937837	G	T	-0.025	1.5E-04
ITGAM	16	cg15817542	rs7499077	A	G	-0.025	1.5E-04
ITGAM	16	cg15817542	rs34594027	C	T	-0.025	1.5E-04
BLK	8	cg21175976	rs2618479	G	A	0.051	1.6E-04
FUS	16	cg16422137	rs41370044	C	T	-0.007	1.6E-04
FUS	16	cg16422137	rs11861251	C	T	-0.007	1.6E-04
PYDC1	16	cg16556397	rs9937837	G	T	0.021	1.7E-04
PYDC1	16	cg16556397	rs7499077	A	G	0.021	1.7E-04
PYDC1	16	cg16556397	rs34594027	C	T	0.021	1.7E-04
IRF5	7	cg26616347	rs11761199	G	A	0.010	1.7E-04
ITGAM	16	cg15817542	rs9925985	C	A	-0.025	1.7E-04
BLK	8	cg21175976	rs1478887	T	C	0.045	1.7E-04
ITGAX	16	cg01486260	rs11150608	A	G	-0.021	1.8E-04
Intergenic	2	cg17648076	rs3024904	A	T	-0.056	1.8E-04
Intergenic	2	cg17648076	rs3024903	T	C	-0.056	1.8E-04
Intergenic	2	cg17648076	rs3024897	C	G	-0.056	1.8E-04
FAM167A	8	cg01383082	rs11250144	C	G	-0.030	1.8E-04
TNIP1	5	cg22178392	rs7734456	C	G	0.013	2.0E-04
IRF5	7	cg00140447	rs3807306	G	T	0.013	2.0E-04
ITGAM	16	cg15817542	rs62051471	T	C	0.026	2.2E-04
Intergenic	8	cg12840336	rs4841558	T	C	-0.018	2.3E-04
Intergenic	8	cg12840336	rs7007439	A	T	-0.018	2.3E-04
IRF5	7	cg00140447	rs11761199	G	A	0.013	2.4E-04
PYDC1	16	cg16556397	rs8061729	C	T	0.022	2.8E-04
PYDC1	16	cg16556397	rs41477949	T	A	0.022	2.8E-04
Intergenic	6	cg01046905	rs169858	C	T	-0.011	2.8E-04
Intergenic	6	cg01046905	rs212395	G	T	-0.011	2.8E-04
Intergenic	6	cg01046905	rs212394	C	G	-0.011	2.8E-04
Intergenic	6	cg01046905	rs212393	A	G	-0.011	2.8E-04
FLNC	7	cg02343182	rs10954214	C	T	0.018	2.9E-04
GPX3	5	cg26891370	rs72790119	T	C	-0.058	3.0E-04
Intergenic	14	cg03549618	rs7155714	A	G	0.053	3.1E-04
Intergenic	14	cg03549618	rs7155561	G	C	0.053	3.1E-04
FAM167A	8	cg02771117	rs11781400	G	T	-0.053	3.2E-04
Intergenic	16	cg03575939	rs41370044	C	T	0.020	3.2E-04
Intergenic	16	cg03575939	rs11861251	C	T	0.020	3.2E-04
Intergenic	16	cg03575939	rs62051472	G	T	0.020	3.2E-04
Intergenic	16	cg03575939	rs62051481	G	A	0.020	3.2E-04
BLK	8	cg21175976	rs2250788	G	A	0.050	3.2E-04
BLK	8	cg21175976	rs998682	G	A	0.050	3.2E-04
BLK	8	cg21175976	rs2618480	C	T	0.050	3.2E-04

IRF5	7	cg26616347	rs10954213	G	A	0.010	3.3E-04
TNFSF4	1	cg24633390	rs3900307	A	C	-0.058	3.5E-04
TNFSF4	1	cg24633390	rs7513384	A	G	-0.058	3.5E-04
ITGAM	16	cg09743950	rs55742763	A	G	0.021	3.5E-04
BLK	8	cg21175976	rs2618451	C	T	0.042	3.6E-04
NA	6	cg01046905	rs212391	T	C	-0.011	3.7E-04
BLK	8	cg24861686	rs73195287	G	C	-0.013	3.7E-04
BLK	8	cg24861686	rs73195289	C	G	-0.013	3.7E-04
BLK	8	cg24861686	rs922485	C	T	-0.013	3.7E-04
BLK	8	cg21175976	rs2618478	A	C	0.050	3.7E-04
BLK	8	cg21175976	rs2250412	A	G	0.049	3.8E-04
TNIP1	5	cg22178392	rs3792794	T	C	0.017	3.9E-04
TNIP1	5	cg22178392	rs3805431	A	G	0.017	3.9E-04
FAM167A	8	cg02771117	rs2618478	A	C	0.066	3.9E-04
TNIP1	5	cg22178392	rs17728260	G	A	0.017	4.3E-04
GPX3	5	cg10802379	rs3763009	A	G	0.012	4.3E-04
FAM167A	8	cg02771117	rs12155745	T	G	-0.052	4.3E-04
Intergenic	6	cg01046905	rs394581	T	C	-0.011	4.4E-04
FLNC	7	cg02343182	rs752637	T	C	0.017	4.5E-04
IRF5	7	cg00140447	rs3807307	T	C	0.012	4.5E-04
Intergenic	2	cg17648076	rs3024900	T	C	-0.054	4.8E-04
TNIP1	5	cg09813400	rs6895271	T	C	-0.015	5.0E-04
IRF5	7	cg00140447	rs10954213	G	A	0.013	5.0E-04
IRF5	7	cg12816198	rs1874328	G	A	0.035	5.1E-04
Intergenic	5	cg26212163	rs2233311	A	C	-0.039	5.3E-04
Intergenic	5	cg26212163	rs72790107	T	C	-0.039	5.3E-04
Intergenic	5	cg26212163	rs2233302	G	C	-0.039	5.3E-04
Intergenic	5	cg26212163	rs72790109	A	G	-0.039	5.3E-04
Intergenic	5	cg26212163	rs72790110	A	G	-0.039	5.3E-04
TNIP1	5	cg09813400	rs34294852	C	T	-0.015	5.4E-04
FAM167A	8	cg01383082	rs12677843	T	C	-0.027	5.4E-04
TNIP1	5	cg09813400	rs3805433	G	C	-0.015	5.6E-04
TNIP1	5	cg09813400	rs12516176	C	T	-0.015	5.6E-04
FAM167A	8	cg01383082	rs17153419	G	A	-0.028	5.7E-04
ITGAM	16	cg08050115	rs55742763	A	G	0.018	5.7E-04
BLK	8	cg24861686	rs73195288	A	G	-0.012	5.7E-04
Intergenic	8	cg12840336	rs10098664	C	T	-0.018	5.7E-04
FAM167A	8	cg15137355	rs1478890	G	C	-0.011	5.9E-04
IRF5	7	cg26616347	rs78658945	A	G	0.013	5.9E-04
BANK1	4	cg01116491	rs6856202	G	A	-0.021	6.1E-04
Intergenic	6	cg13362513	rs115837294	T	G	-0.047	6.2E-04
PYCARD	16	cg07461837	rs2359661	G	A	-0.014	6.2E-04
STAT1	2	cg25856179	rs4853457	T	A	0.004	6.3E-04
TNIP1	5	cg18443811	rs3763009	A	G	0.006	6.3E-04
PYCARD	16	cg07461837	rs11150608	A	G	-0.015	6.6E-04
FAM167A	8	cg02771117	rs13272061	T	G	-0.054	7.0E-04
Intergenic	16	cg03575939	rs62051471	T	C	-0.016	7.1E-04
FAM167A	8	cg02771117	rs13275864	G	C	-0.050	7.2E-04
KCP	7	cg11461236	rs752637	T	C	0.010	7.5E-04
BLK	8	cg24861686	rs922484	C	T	-0.011	7.6E-04
FCGR2A	1	cg06350097	rs10494360	A	G	-0.054	7.7E-04
FCGR2A	1	cg06350097	rs9427399	C	T	-0.054	7.8E-04
FCGR2A	1	cg06350097	rs7515174	G	C	-0.054	7.8E-04

FCGR2A	1	cg06350097	rs9427400	T	C	-0.054	7.8E-04
FCGR2A	1	cg06350097	rs6671753	A	G	-0.054	7.8E-04
FCGR2A	1	cg06350097	rs7529225	A	G	-0.054	7.8E-04
FCGR2A	1	cg06350097	rs7552317	T	C	-0.054	7.8E-04
FCGR2A	1	cg06350097	rs7529425	A	G	-0.054	7.8E-04
FCGR2A	1	cg06350097	rs11810143	G	A	-0.054	7.8E-04
FAM167A	8	cg15137355	rs12155745	T	G	-0.011	7.8E-04
ITGAX	16	cg01486260	rs11641202	G	A	-0.018	8.1E-04
ITGAM	16	cg02846316	rs41370044	C	T	0.013	8.1E-04
ITGAM	16	cg02846316	rs11861251	C	T	0.013	8.1E-04
ITGAM	16	cg02846316	rs62051472	G	T	0.013	8.1E-04
ITGAM	16	cg02846316	rs62051481	G	A	0.013	8.1E-04
FCGR2A	1	cg06350097	rs9427398	G	A	-0.052	8.3E-04
BANK1	4	cg01116491	rs7683892	T	C	-0.021	8.4E-04
BANK1	4	cg01116491	rs10031210	A	G	-0.021	8.4E-04
BANK1	4	cg01116491	rs7690997	G	C	-0.021	8.4E-04
BANK1	4	cg01116491	rs7691030	C	T	-0.021	8.4E-04
BANK1	4	cg01116491	rs77613358	C	T	-0.021	8.4E-04
BANK1	4	cg01116491	rs80241059	G	A	-0.021	8.4E-04
BANK1	4	cg01116491	rs4522865	G	A	-0.021	8.4E-04
BANK1	4	cg01116491	rs7656409	A	G	-0.021	8.4E-04
BANK1	4	cg01116491	rs7656627	A	G	-0.021	8.4E-04
BANK1	4	cg01116491	rs7699179	T	C	-0.021	8.4E-04
BANK1	4	cg01116491	rs6833764	G	A	-0.021	8.4E-04
Intergenic	1	cg23270415	rs4656308	T	C	-0.015	8.4E-04
BLK	8	cg21175976	rs2252729	G	A	0.053	8.9E-04
BLK	8	cg21175976	rs2618458	G	A	0.053	8.9E-04
BLK	8	cg21175976	rs2618457	G	A	0.053	8.9E-04
TNIP1	5	cg09813400	rs12518386	A	G	-0.014	9.0E-04
Intergenic	12	cg05909891	rs10841216	G	A	-0.017	9.0E-04
FAM167A	8	cg15137355	rs17153419	G	A	0.010	9.1E-04
NOS1	12	cg02962597	rs561712	T	C	0.047	9.1E-04
ATP6V1F	7	cg26682717	rs10954214	C	T	0.009	9.2E-04
ITGAX	16	cg01486260	rs2359661	G	A	-0.017	9.3E-04
IRF5	7	cg12816198	rs752637	T	C	-0.032	9.3E-04

All meQTLs with p<0.001 are shown. meQTL: methylation quantitative locus. Chr: Chromosome. The slope refers to the effect of the minor allele for the methylation beta value at a given CpG site compared to the major allele.
